# Discovery of Dual TRPA1 and TRPV1 Antagonists as Novel Therapeutic Agents for Pain

**DOI:** 10.3390/ph17091209

**Published:** 2024-09-13

**Authors:** Nayeon Do, Dongxu Zuo, Miri Kim, Minseok Kim, Hee-Jin Ha, Peter M. Blumberg, Jihyae Ann, Sun Wook Hwang, Jeewoo Lee

**Affiliations:** 1 College of Pharmacy, Seoul National University, Seoul 08826, Republic of Korea; nydoh@snu.ac.kr (N.D.); dongxu_zuo@outlook.com (D.Z.); burrows@fred.net (P.M.B.); jihuya@snu.ac.kr (J.A.); 2 Department of Biomedical Sciences, Korea University College of Medicine, Seoul 02841, Republic of Korea; mrkim403@korea.ac.kr (M.K.); minseok7929@korea.ac.kr (M.K.); 3 Medifron DBT, Seoul 08502, Republic of Korea; hjha@n-dic.com

**Keywords:** TRPA1, TRPV1, dual antagonist, analgesic

## Abstract

Pain management remains a major challenge in medicine, highlighting the need for the development of new therapeutic agents. The transient receptor potential ankyrin 1 (TRPA1) and vanilloid 1 (TRPV1) are ion channels that play key roles in pain perception. Targeting both TRPA1 and TRPV1 simultaneously with dual antagonists offers a promising approach to pain relief. In this study, we investigated a series of hybrid analogs of TRPA1 and TRPV1 antagonists to discover novel therapeutic agents for pain. Among these compounds synthesized by a condensation reaction forming 1,2,4-oxadiazole between the A- and C-regions, compound **50** exhibited substantial dual-acting antagonism to TRPA1 and TRPV1 with IC_50_ values of 1.42, 2.84, 2.13, and 5.02 μM for hTRPA1, mTRPA1, hTRPV1, and rTRPV1, respectively. In the formalin test, compound **50** demonstrated dose-dependent analgesic activity with an ED_50_ of 85.9 mg/kg in phase 1 and 21.6 mg/kg in phase 2, respectively, and was able to inhibit pain behavior completely at a dose of 100 mg/kg. This study presents the discovery and characterization of a novel dual TRPA1/TRPV1 antagonist, highlighting its potential as a therapeutic agent for pain management.

## 1. Introduction

Pain is a multifaceted physiological response typically linked to tissue damage and inflammation. Traditional pain medications, such as opioids and NSAIDs, frequently come with considerable side effects and limitations, underscoring the urgent need for more effective and safer pain relief options [1].

**Figure 1:**
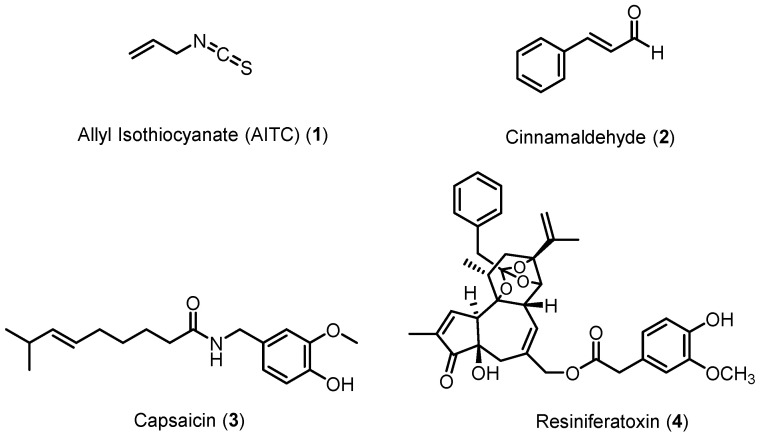
TRPA1 and TRPV1 agonists.

The transient receptor potential ankyrin 1 (TRPA1) is a calcium-permeable non-selective cation channel that forms a unique branch within the TRP family of ion channels. The channel can be activated by exogenous irritants, such as allylisothiocyanate (**1**, AITC), cinnamaldehyde (**2**), allicin, 4-hydroxynonenal and mediators produced by inflammation or tissue injury, as well as by cold and mechanical stimuli (Figure 1) [2]. TRPA1 is expressed in sensory neurons and co-localizes with TRPV1, calcitonin gene-related peptide, substance P and neurokinin-A. TRPA1 acts as a molecular integrator of various stimuli to mediate sensation, pain, and neurogenic inflammation [3]. Therefore, the drug development of TRPA1 antagonists has been extensively pursued for the treatment of pain, inflammation, and related diseases [4].

The transient receptor potential vanilloid member 1 (TRPV1) ion channel is also a non-selective cation channel with high Ca^2+^ permeability that is activated by endogenous factors such as low pH, elevated temperature, and endovanilloids, as well as by natural vanilloids such as capsaicin (**3**) and resiniferatoxin (**4**) (Figure 1). Upon stimulation, receptor activation leads to an increase in intracellular Ca^2+^ that results in the excitation of primary sensory neurons and, ultimately, in the central perception of pain, itching, and burning. Its pivotal role in the pain pathway has garnered significant attention as a therapeutic target for pain [5,6], sparking interest in other members of the TRP family [7,8,9,10].

TRPA1 and TRPV1 are structurally related thermosensitive cation channels co-expressed in nociceptive primary sensory neurons and play an integrative role in regulating pain processing and inflammatory functions [11]. Both receptors contribute to inflammatory and neuropathic pain through multiple mechanisms, including upregulation and sensitization via a variety of exogenous stimuli, endogenous inflammatory mediators, and metabolites of oxidative stress [12]. In addition, both channels have some interactions with the endocannabinoid system [13]. Therefore, the combined pharmacological intervention at TRPA1 and TRPV1 could provide a novel strategy for treating related painful diseases. 

It has been demonstrated that the modulation of both TRPA1 and TRPV1 was very effective in controlling a variety of preclinical pain models, including inflammatory pain [14], cancer pain [15], radiotherapy-related pain [16], chronic pelvic pain [17], orthodontic pain [18], muscle pain [19], burn injury-induced chronic pain [20], and inflammatory models including lung inflammation [21,22,23,24], bowel inflammation [25], skin inflammation [26], and pancreatic inflammation [27]. In addition to a direct role in pain and neurogenic inflammation, both receptors are also expressed on a multitude of non-neuronal sites, from vascular smooth muscle to keratinocytes and endothelium, in which they play diverse roles in pathophysiology [28]. Among other indications, the dual inhibition of TRPA1 and TRPV1 expression has the potential for controlling itching [29,30] and eye allergy [31]. Taken together, a growing body of evidence indicates that dual inhibition of TRPA1 and TRPV1 may represent a primary therapeutic target for relieving diverse painful conditions, as well as treating inflammatory and other non-neuronal diseases. 

Clinical development of TRPV1 antagonists for systemic application was largely halted because the drugs caused hyperthermia and put patients at risk for scalding injuries by elevating the heat pain threshold [32]. On the other hand, TRPA1-targeted drug development suffered from pharmaceutical and pharmacokinetic complications. Thus, it would be intriguing to discover dual-acting antagonists with potential antinociceptive properties that are associated with TRPV1 and TRPA1 modulation. The identification of such ligands could lead to the analgesic candidate with enhanced pain-relieving effects and reduced side effects compared to a single-mechanism antagonist.

**Figure 2:**
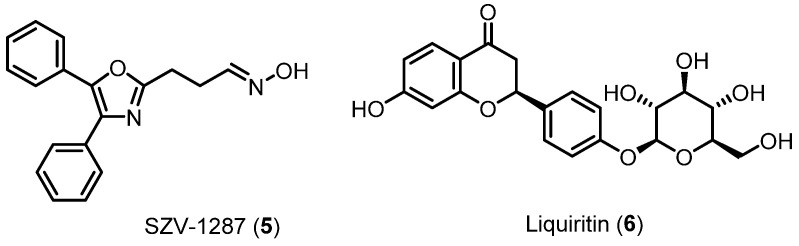
Dual TRPA1 and TRPV1 antagonists.

A few dual antagonists have been reported, and their studies have supported proof of concept in preclinical models. SZV-1287 (3-(4,5-diphenyl-1,3-oxazol-2-yl)propanal oxime) is a potent dual antagonist of TRPA1 and TRPV1 ion channels on primary sensory neurons, exerting significant concentration-dependent inhibition on both AITC- and capsaicin-evoked calcium influx in trigeminal neurons and in TRPA1 or TRPV1 receptor-expressing cell lines (Figure 2) [33]. Acute SZV-1287 administration resulted in approximately 50% significant reduction of neuropathic hyperalgesia after nerve ligation, which was not observed in either TRPA1- or TRPV1-deficient mice [34]. In addition, SZV-1287 exerted potent analgesic and anti-inflammatory actions in various chronic arthritis models without inducing cartilage damage [35]. In a proof-of-concept study, an oral capsule formulation of SZV 1287 inhibited neuropathic mechanical hyperalgesia in a partial sciatic nerve ligation-induced traumatic neuropathy model, and despite TRPV1 antagonistic activity and brain penetration, it did not substantially influence thermoregulation in mice [36].

Liquiritin, a flavonoid ingredient of licorice, is a dual-acting antagonist that inhibits capsaicin- and allyl isothiocyanate-evoked TRPV1 and TRPA1 activation with similar potencies and maximal inhibition, respectively (Figure 2) [37]. In a mouse acute lung injury (ALI) model involving both TRPV1 and TRPA1, an oral gavage of liquiritin prevented tissue damage and suppressed inflammation and the activation of the NF-κB signaling pathway in the lung tissue. Liquiritin also suppressed LPS-induced increase in TRPV1 and TRPA1 protein expression in the lung tissue, as well as TRPV1 and TRPA1 mRNA levels in cells contained in mouse bronchoalveolar lavage fluid. A target engagement study using capsazepine and HC030031 demonstrated that the anti-inflammatory and antitussive effects of liquiritin are mediated by the dual inhibition of TRPV1 and TRPA1 channels, which are upregulated in non-neuronal cells through the NF-κB pathway during airway inflammation via a positive feedback mechanism.

Compound **7** (AM-0902) is a potent and selective TRPA1 antagonist with favorable in vivo pharmacokinetic properties [38]. It showed dose-dependent and nearly complete inhibition of AITC-induced flinching in rats upon oral administration. Compound **8** is a potent TRPV1 antagonist in rat dorsal root ganglia neurons with an IC_50_ = 1 nM [39]. It displayed an anti-hyperalgesic effect, showing a 60% reversion of CFA-induced tactile allodynia after oral administration of 30 μmol/kg in rats.

**Figure 3:**
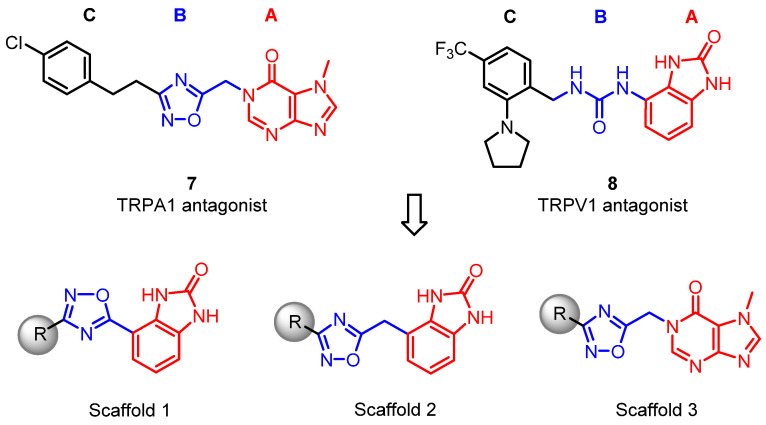
Design of dual-acting TRPA1 and TRPV1 antagonists.

On the basis of previous pharmacophore analyses, the pharmacophores of both compounds **7** and **8** can be partitioned by A, B, and C regions, respectively (Figure 3), in which the TRPA1 and TRPV1 antagonist appears to share similar pharmacophores.

To design a compound that dually antagonizes TRPA1 and TPRV1, a strategy of merging the pharmacophore of the two targets was utilized. The merged-pharmacophore scaffold contains the following features (Figure 3). First, the B-region was fixed with 1,2,4-oxadiazole instead of the urea moiety since the latter moiety causes solubility issues. In the A-region, the purinone of compound **7** or the benzimidazolone of compound **8** was introduced. In the C-region, the 4-chlorophenethyl and biphenyl groups of the TRPA1 C-region and the 2-(trifluoromethyl)pyridine, 2-(trifluoromethyl or *t*-butyl)thiazole, and 3-(*t*-butyl)pyrazole derivatives of the TRPV1 C-region libraries constructed in the course of prior research in our laboratory were utilized. Based on this strategy, 31 new compounds of the three scaffolds were synthesized by the reaction forming 1,2,4-oxadiazole between the A- and C-region and evaluated for antagonistic activity toward rat and human TRPA1 and TRPV1.

## 2. Results and Discussion

### 2.1. Synthesis

Generally, the 1,2,4-oxadiazole target compounds were synthesized by the condensation between the carboxylic acid of the A-region and the corresponding *N*-hydroxy-imidamide of the C-region.

**Scheme 1:**
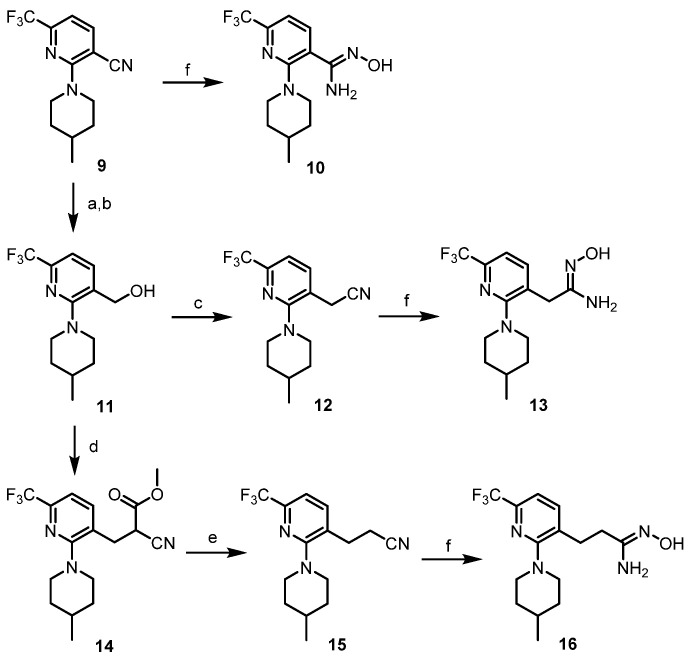
Synthesis of *N*-hydroxy-imidamides of the pyridine C-region. **Reagents and conditions**: (a) KOH, ethylene glycol, reflux, 24 h; (b) LiAlH_4_, diethylether, 0 °C to r.t., 2 h; (c) MsCl, NEt_3_, CH_2_Cl_2_, 0 °C to r.t., 2 h, then KCN, DMSO, r.t., 2 h; (d) MsCl, NEt_3_, CH_2_Cl_2_, 0 °C to r.t., 1 h, then NaH, methylcyanoacetate, DMF, 0 °C to r.t., 3 h; (e) LiCl, DMSO/H_2_O (3:1), 100 °C, 24 h; and (f) NH_2_OH, EtOH, 50 °C, 1–4 h.

For the synthesis of *N*-hydroxy-imidamides of the pyridine C-region (Scheme 1), the previously reported nicotinonitrile (**9**) [40] was hydrolyzed and then reduced to afford the corresponding alcohol (**11**), which was converted to the one-carbon elongated nitrile (**12**). Meanwhile, the alcohol (**11**) was mesylated and then reacted with methylcyanoacetate to provide **14**, which underwent Krapcho decarboxylation [41] by lithium chloride to yield the two-carbon elongated nitrile (**15**). The nitriles (**9**, **12**, **15**) were reacted with hydroxylamine to provide the corresponding *N*-hydroxy-imidamides (**10**, **13**, **16**), respectively.

**Scheme 2:**
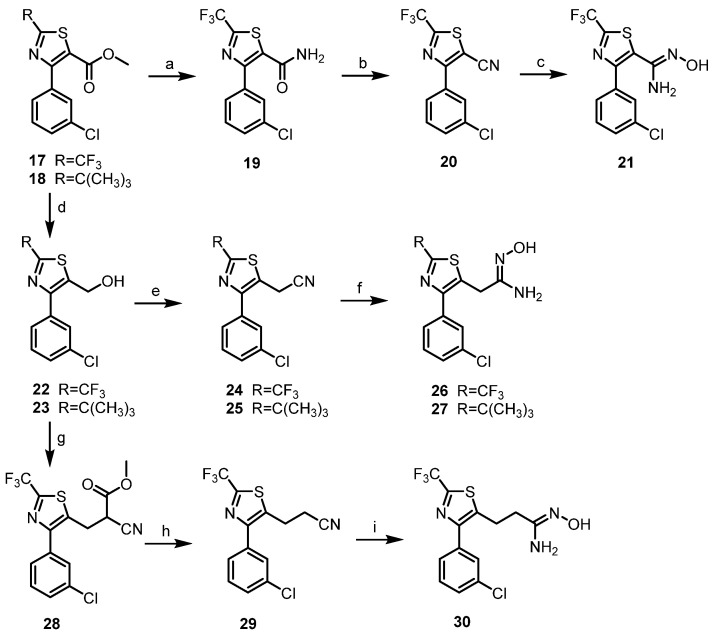
Synthesis of *N*-hydroxy-imidamides of the thiazole C-region. **Reagents and conditions**: (a) NH_3_, MeOH, 60 °C, 15 h; (b) TFAA, pyridine, THF, 0 °C to r.t., 1 h; (c) NH_2_OH, EtOH, 50 °C, 1 h; (d) LiAlH_4_, diethylether, 0 °C, 15 min; (e) MsCl, NEt_3_, CH_2_Cl_2_, 0 °C to r.t., 1 h, then KCN, DMSO, r.t., 20 min; (f) NH_2_OH, EtOH, 50 °C, 2 h; (g) MsCl, NEt_3_, CH_2_Cl_2_, 0 °C to r.t., 1 h, then NaH, methyl cyanoacetate, DMF, 0 °C to r.t., 3 h; (h) LiCl, DMSO/H_2_O (3:1), 100 °C, 24 h; and (i) NH_2_OH, EtOH, 50 °C, 4 h.

For the synthesis of *N*-hydroxy-imidamides of the thiazole C-region (Scheme 2), the methyl ester of the thiazole intermediate (**17**) previously reported [42] was converted to the corresponding nitrile (**20**) via the amide, which was reacted with hydroxylamine to give the corresponding *N*-hydroxy-imidamide (**21**). On the other hand, methyl esters (**17**, **18**) were reduced to the corresponding alcohols, which were transformed to the one- (**26**, **27**) and two-carbon elongated *N*-hydroxy-imidamides (**30**), respectively, by following the same method used in Scheme 1.

**Scheme 3:**
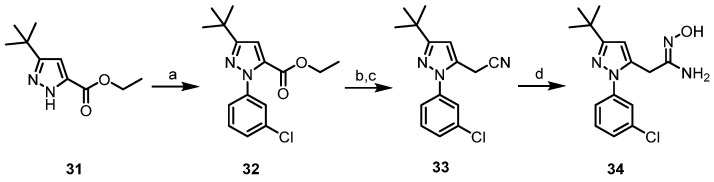
Synthesis of *N*-hydroxy-imidamides of the pyrazole C-region. **Reagents and conditions**: (a) (3-chlorophenyl)boronic acid, Cu(OAc)_2_, pyridine, CH_2_Cl_2_, r.t., 22 h; (b) LiAlH_4_, diethylether, 0 °C, 15 min; (c) MsCl, NEt_3_, CH_2_Cl_2_, 0 °C to r.t., 1 h, then KCN, DMSO, r.t., 20 min; (d) NH_2_OH, EtOH, 50 °C, 2 h.

For the synthesis of *N*-hydroxy-imidamides of the pyrazole C-region (Scheme 3), the pyrazole (**31**) previously reported [43] was reacted with (3-chlorophenyl)boronic acid by Chan-lam coupling [44] to provide 3-chlorophenylpyrazole (**32**), whose ester was converted to the corresponding *N*-hydroxy-imidamides (**34**) employing the same procedures above.

**Scheme 4:**
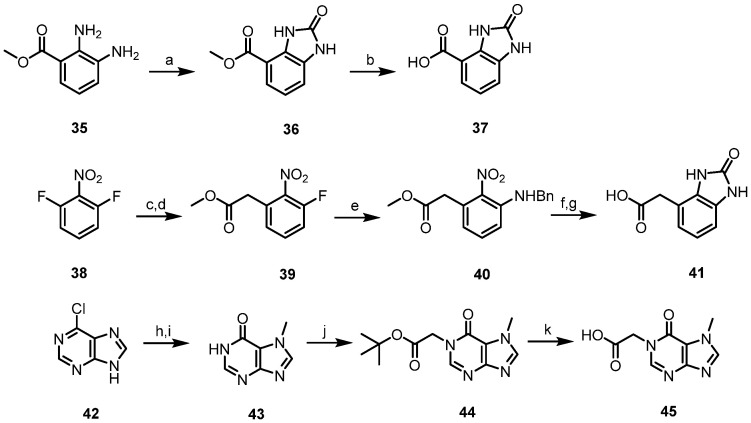
Synthesis of carboxylic acid of benzimidazolone and purinone A-regions. **Reagents and conditions**: (a) CDI, Et_3_N, THF, reflux, 21 h; (b) NaOH, MeOH/H_2_O (2:1), r.t., 16 h; (c) NaH, dimethylmalonate, DMF, 0 °C to 50 °C, 15 h; (d) LiCl, DMSO/H_2_O (3:1), 100 °C, 18 h; (e) benzylamine, Et_3_N, DMF, 60 °C, 13 h; (f) Pd/C, H_2_, MeOH, r.t., 1 h, then CDI, THF, r.t., 1 h; (g) NaOH, MeOH/H_2_O (2:1), r.t., 2 h; (h) MeMgCl, MeI, anhyd.THF, 50 °C, 15 h; (i) HCO_2_H, EtOH, 75 °C to reflux, 3 h; (j) *t*-butyl bromoacetate, K_2_CO_3_, DMF, 50 °C, 6 h; and (k) c-HCl, r.t., 30 min.

For the synthesis of the carboxylic acid of the benzimidazolone A-region (Scheme 4), methyl 2,3-diaminobenzoate (**35**) was cyclized to form the benzimidazolone (**36**) using carbonyldiimidazole (CDI), which was hydrolyzed to the acid (**37**). On the other hand, 1,3-difluoro-2-nitrobenzene (**38**) was condensed with dimethylmalonate followed by Krapcho decarboxylation to yield methyl ester **39**. The benzylamine substitution of **39** was followed by catalytic reduction to provide the diamine, which was cyclized by CDI to provide the one-carbon elongated acid of the benzimidazolone A-region (**41**).

For the synthesis of the carboxylic acid of the purinone A region (Scheme 4), 6-chloropurine (**42**) was selectively methylated at the 7 position using methylmagnesium chloride as a base and then oxidized under formic acid to yield the 7-methylpurin-6-one (**43**). Purinone 41 was alkylated with *t*-butyl bromoacetate and then hydrolyzed under acidic conditions to provide the carboxylic acid of the purinone A-region (**45**).

**Scheme 5:**
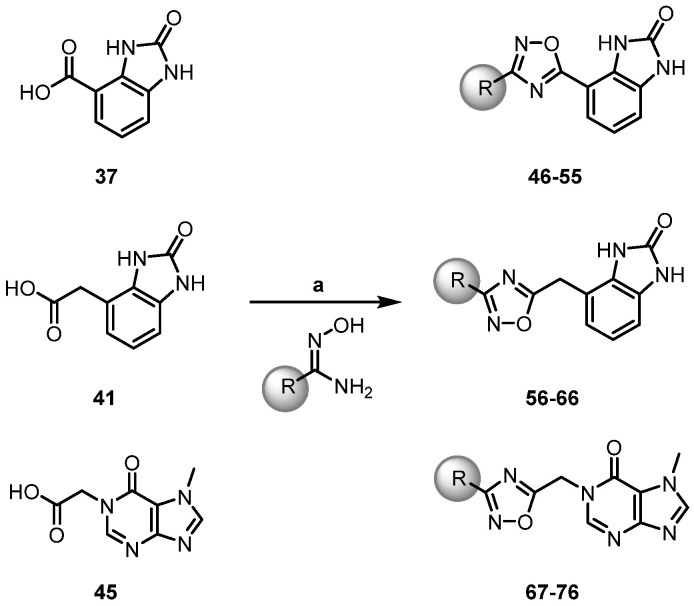
**Reagents and conditions**: (a) CDI, DMF, 50 °C to 100 °C, 15–24 h.

Finally, the 1,2,4-oxadiazole target compounds were synthesized by the condensation between the prepared *N*-hydroxy-imidamides of the C-region and the carboxylic acid of the A-region using CDI to provide the final compounds (**46**–**76**) (Scheme 5).

### 2.2. Biological Activity

#### 2.2.1. In Vitro Activity

The in vitro assay for TRPA1 and TRPV1 antagonism was performed using a fluorometric imaging plate reader (FLIPR) with Chinese hamster ovary (CHO) cells expressing *h*TRPV1 and human embryonic kidney (HEK293) cells expressing *h*TRPA1, *m*TRPA1 and *r*TRPV1. The antagonistic activity of the synthesized compounds was measured by inhibition of TRPA1 activation by cinnamaldehyde (100 µM) and TRPV1 activation by capsaicin (1 µM), respectively. The inhibitory activity was expressed as percent inhibition using a concentration of 10 μM compound along with the reference compounds, AM-0902 (TRPA1 antagonist) and BCTC (TRPV1 antagonist). The results are summarized in Table 1, Table 2 and Table 3.

In the first scaffold, the A- and B-regions were fixed with benzimidazolone and 1,2,4-oxadiazole, respectively, and then the activity was analyzed as the C-region was varied (Table 1). The 4-chlorophenylethyl analog (**46**) exhibited moderate antagonism toward TRPA1 activation and weak inhibition toward TRPV1 activation. The biphenyl analog (**47**) showed similar activity to that of **46**, whereas its geometric isomer (**48**) displayed favorable activity only toward TRPV1. The 6-trifluoro-2-(4-methyl-piperdin-1-yl)pyridine C-region analogs (**49**–**51**), which were employed in TRPV1 antagonists, were explored by varying the length of the linker between the oxadiazole and pyridine. Among them, compound **50** with a one-carbon linker demonstrated significant inhibitory activity on both channels, with 60% inhibition of *h*TRPA1 activation and 52% inhibition of *h*TRPV1 activation. The thiazole analogs were also explored. The 2-trifluoromethyl thiazole analogs (**52**, **53**) showed inhibitory activity only on mTRPA1, whereas the 2-*t*-butyl thiazole analog (**54**) displayed significant inhibition only on hTRPV1. The 3-*t*-butyl pyrazole analog (**55**) demonstrated significant activity towards TRPA1 but not towards TRPV1.

In the second scaffold, one carbon was elongated between the A- and B-regions of the first scaffold (Table 2). The 4-chlorophenylethyl analog (**56**) exhibited better antagonism toward hTRPV1 compared to that of **46**. The two biphenyl analogs (**57**, **58**) demonstrated moderate inhibition on both channels, similar to **47** and **48**. The same pyridine C-region analogs (**59**–**61**), as shown in Table 1, were examined. Among them, compound **59** without a linker showed favorable antagonism to TRPA1, whereas compound **61** with the two-carbon linker displayed favorable antagonism to TRPV1 compared to compound **60**. The thiazole analogs (**62**–**65**) provided favorable antagonism to TRPV1, and in particular, compound **65** displayed significant inhibition in TRPV1 with 69% inhibition of hTRPV1. The pyrazole analog (**66**) displayed substantial inhibition to both receptors.

In the third scaffold, the A- and B-region was used with 7-methyl-1,7-dihydro-6H-purin-6-one and 1,2,4-oxadiazole as in AM-0902, and the C-region was modified using that of a TRPV1 antagonist (Table 3). In the biphenyl analogs, compound **67** did not show inhibition to both channels, whereas compound **68** displayed moderate inhibition. Most pyridine, thiazole, and pyrazole analogs (**69**–**76**) showed weak inhibition on both channels in which compounds **72**, **75** and **76** exhibited more than 40% inhibition only to mTRPA1. The SAR analysis indicated that benzimidazolone A-region analogs were more active than the corresponding purinone A-region analogs in *h*TRPA and *h*TRPV1 inhibition.

**Figure 4:**
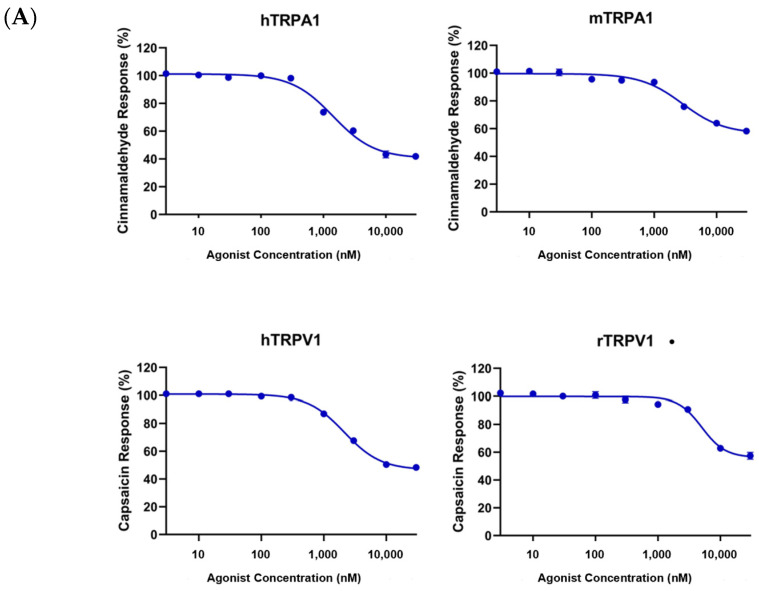

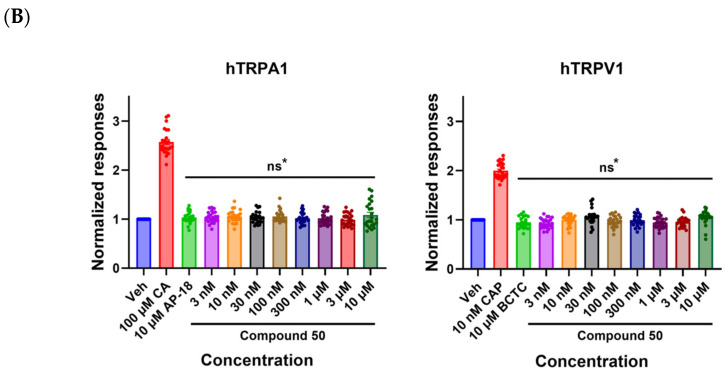
Functional activities of compound **50** toward TRPA1 and TRPV1. (**A**) Concentration-dependent antagonistic activities toward hTRPA1, mTRPV1, hTRPV1, and rTRPV1. (**B**) Agonistic activities toward hTRPA1 and hTRPV1. * ns: not significant, *p* > 0.05.

Overall, considering significant dual inhibition on both channels, compound **50** was selected for further investigation for concentration-dependent inhibition. The inhibitory activity of compound **50** was measured on both channels in a concentration-dependent manner, resulting in IC_50_ values of 1.42, 2.84, 2.13, and 5.02 μM for hTRPA1, mTRPA1, hTRPV1 and rTRPV1, respectively (Figure 4A). The compound did not exhibit complete inhibition at concentrations up to 30 μM but did not exhibit any agonism at the concentrations tested (Figure 4B). We conclude that compound **50,** in effect, induces partial antagonism, which is able to inhibit the action of the agonist but is unable to block receptor activity completely. Its behavior is not consistent with so-called partial agonism but reflects some other mechanism, such as limiting solubility.

#### 2.2.2. In Vivo Activity

To evaluate the antinociceptive activity of compound **50**, we conducted the formalin test in ICR mice [45,46]. In this test, the compound was administered by intraperitoneal (ip) injection 30 min before subcutaneous (sc) injection of 2% formalin solution into the hind paw, and then the pain response of licking and biting of the injected paw was evaluated over the next 30 min. The evaluation was performed over two discrete phases (phase 1, 0–5 min; phase 2, 15–30 min) of the response upon formalin injection. Phase 1 (acute phase) is characterized by intense pain and is believed to be due to direct chemical stimulation of nociceptors, whereas phase 2 (chronic phase) is thought to reflect ongoing inflammation and central sensitization.

**Figure 5:**
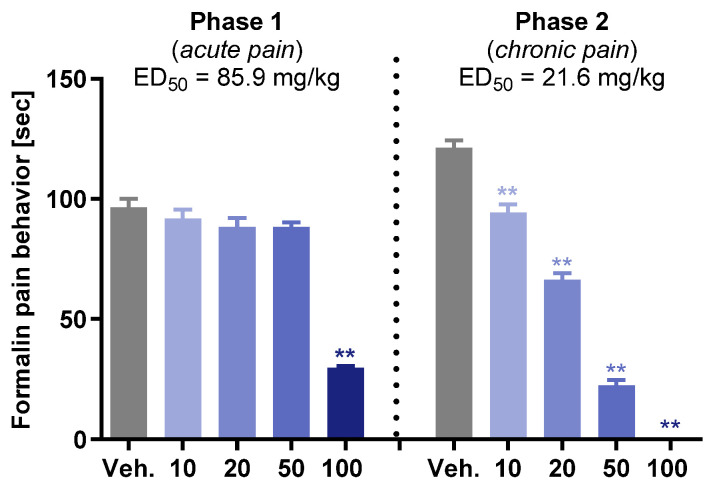
In vivo analgesic activity of compound **50** in formalin model. Results are expressed as mean ± SEM (*n* = 4). ** *p* < 0.01 compared to the vehicle-treated group.

As shown in Figure 5, compound **50** exhibited promising antinociceptive activity in a dose-response fashion with an ED_50_ of 85.9 mg/kg in phase 1 and 21.6 mg/kg in phase 2, respectively. Additionally, at a dose of 100 mg/kg, compound **50** was able to completely inhibit pain behavior in phase 2 without any observed toxicity.

## 3. Materials and Methods

### 3.1. Chemistry

All chemical reagents and solvents were commercially available. Silica gel column chromatography was performed using ZEOprep 60/40–63 μm silica gel (ZEOCHEM, Louisville, KY, USA). ^1^H and ^13^C NMR spectra were recorded on JEOL JNM-ECZ400S spectrrometer (400 MHz for ^1^H and 100 MHz for ^13^C; JEOL Ltd., Akishima, Tokyo, Japan). Chemical shifts are reported in parts per million (ppm) relative to tetramethylsilane (Me_4_Si) as internal standard. High-resolution mass spectra (HRMS) were measured by fast atom bombardment (FAB) with a JEOL JMS-700 MStation instrument (JEOL Ltd., Akishima, Tokyo, Japan). All final compounds were purified to greater than 95% purity, as determined by high-performance liquid chromatography (HPLC). HPLC was performed on an Agilent 1120 Compact LC (G4288A) instrument (Agilent Technologies, Santaclara, CA, USA) using an Agilent TC-C18 column (4.6 mm × 250 mm, 5 μm).

#### 3.1.1. General Procedure for Reduction (Procedure A)

Lithium aluminum hydride (2.0 equiv.) was slowly added to a solution of ester (1.0 equiv.) in THF or diethyl ether at 0 °C. The reaction mixture was allowed to warm to room temperature for 15 min–2 h. After completion, the reaction mixture was quenched with water and extracted with CH_2_Cl_2_. The organic layer was dried over MgSO_4_ and concentrated in vacuo. The residue was purified by column chromatography on silica gel to afford the desired compound.

#### 3.1.2. General Procedure for Cyanation (Procedure B)

Triethylamine (1.5 equiv.) and methanesulfonyl chloride (1.5 equiv.) were added to a solution of alcohol (1.0 equiv.) in CH_2_Cl_2_ at 0 °C and the mixture was stirred for 1–2 h. The reaction mixture was partitioned between CH_2_Cl_2_ and water. The organic layer was washed with water, dried over MgSO_4_, and concentrated in vacuo. Potassium cyanide (2.0 equiv.) was added to a solution of obtained residue in DMSO. The mixture was stirred for 20 min–2 h at room temperature. The reaction mixture was quenched with water and extracted with EtOAc. The organic layer was dried over MgSO_4_, and concentrated in vacuo. The residue was purified by column chromatography on silica gel to afford the desired compound.

#### 3.1.3. General Procedure for Preparation of N’-Hydroxy-Imidamide (Procedure C)

Hydroxylamine 50% aq. soln (4.0 equiv.) was added to a solution of nitrile (1.0 equiv.) in ethanol (3.8 mL/mmol). The reaction mixture was heated to 50 °C. After 1–4 h, the solvent was concentrated in vacuo. The residue was purified by column chromatography on silica gel to afford the desired compound.

#### 3.1.4. General Procedure for Formation of Cyano Ester (Procedure D)

To a solution of alcohol (1.0 equiv.) in CH_2_Cl_2_ were added triethylamine (1.5 equiv.) and methanesulfonyl chloride (1.5 equiv.) at 0 °C and the mixture was stirred for 1 h. The reaction mixture was partitioned between CH_2_Cl_2_ and water. The organic layer was washed with water, dried over MgSO_4_, and concentrated in vacuo. The obtained residue was dissolved in DMF, and 60% sodium hydride was added portion-wise while dispersing it in paraffin liquid (1.5 equiv.) at 0 °C. After 10 min, methylcyanoacetate (1.5 equiv.) was added, and the reaction mixture was stirred for 3 h at room temperature. The reaction mixture was quenched with water and extracted with EtOAc. The organic layer was dried over MgSO_4_ and concentrated in vacuo. The residue was purified by column chromatography on silica gel to afford the desired compound.

#### 3.1.5. General Procedure for Krapcho Decarboxylation (Procedure E)

To a solution of cyano ester or malonate (1.0 equiv.) in DMSO/H_2_O (3:1) was added lithium chloride (3.0 equiv.) and the mixture was stirred at 100 °C for 18–24 h. The reaction mixture was quenched with water and extracted with EtOAc. The organic layer was dried over MgSO_4_, and concentrated in vacuo. The residue was purified by column chromatography on silica gel to afford the desired compound.

#### 3.1.6. General Procedure for Hydrolysis (Procedure F)

Sodium hydroxide (2.0 equiv.) was added to a solution of ester (1.0 equiv.) in MeOH/H_2_O (2:1), and the mixture was stirred for 2–16 h at room temperature. The pH of the resulting mixture was adjusted to pH 2 using 1 N HCl and extracted with EtOAc. The organic layer was dried over MgSO_4_, and concentrated in vacuo to afford the desired compound, which was used in the next step without further purification

#### 3.1.7. General Procedure for Condensation of 1,2,4-Oxadiazole (Procedure G)

1,1′-carbonyldiimidazole (1.2 equiv.) was added to a solution of carboxylic acid (1.1 equiv.) in DMF, and the mixture was stirred at 50 °C for 30 min. N’-hydroxy-imidamide (1.0 equiv.) was then added, and the mixture was stirred at 100 °C for 15–24 h. The reaction mixture was quenched with water and extracted with EtOAc. The organic layer was dried over MgSO_4_ and concentrated in vacuo. The residue was purified by column chromatography on silica gel to afford the desired compound.

*N’-Hydroxy-2-(4-methylpiperidin-1-yl)-6-(trifluoromethyl)nicotinimidamide* (**10**). Compound **10** was synthesized from **9** according to general procedure C. 62% yield, pale yellow oil; ^1^H NMR (400 MHz, DMSO-*d*_6_) *δ* 9.56 (s, 1H), 7.65 (d, *J* = 7.8 Hz, 1H), 7.14 (d, *J* = 7.6 Hz, 1H), 5.83 (s, 2H), 3.88 (d, *J* = 12.8 Hz, 2H), 2.74 (t, *J* = 11.4 Hz, 2H), 1.59 (d, *J* = 12.8 Hz, 2H), 1.47–1.54 (m, 1H), 1.13–1.23 (m, 2H), 0.88 (d, *J* = 6.4 Hz, 3H).
*(2-(4-Methylpiperidin-1-yl)-6-(trifluoromethyl)pyridin-3-yl)methanol* (**11**). Potassium hydroxide (8.0 equiv.) was added to a solution of **9** (1.0 equiv.) in ethylene glycol and the mixture was refluxed for 24 h. After completion of the reaction, the mixture was cooled to 0 °C and diluted with water. The pH of the resulting mixture was adjusted to pH 2 using 1 N HCl and extracted with CH_2_Cl_2_. The organic layer was dried over MgSO_4_, and concentrated in vacuo. Compound **11** was synthesized from the obtained residue according to general procedure A. 66% yield, pale yellow oil; ^1^H NMR (400 MHz, CDCl_3_) *δ* 7.71 (d, *J* = 7.6 Hz, 1H), 7.30 (d, *J* = 7.2 Hz, 1H), 4.75 (s, 2H), 3.37 (d, *J* = 12.8 Hz, 2H), 2.89 (td, *J* = 12.4, 2.3 Hz, 2H), 1.77 (d, *J* = 10.8 Hz, 2H), 1.54–1.62 (m, 1H), 1.35 (qd, *J* = 12.4, 3.6 Hz, 2H), 0.99 (d, *J* = 6.4 Hz, 3H).
*2-(2-(4-Methylpiperidin-1-yl)-6-(trifluoromethyl)pyridin-3-yl)acetonitrile* (**12**). Compound **12** was synthesized from **11** according to general procedure B. 96% yield, pale yellow oil; ^1^H NMR (400 MHz, CDCl_3_) *δ* 7.86 (d, *J* = 7.8 Hz, 1H), 7.32 (d, *J* = 7.8 Hz, 1H), 3.76 (s, 2H), 3.28 (d, *J* = 13.3 Hz, 2H), 2.88 (td, *J* = 12.6, 2.1 Hz, 2H), 1.76 (d, *J* = 12.8 Hz, 2H), 1.53–1.62 (m, 1H), 1.34 (qd, *J* = 12.2, 3.7 Hz, 2H), 1.00 (d, *J* = 6.4 Hz, 3H).
*N’-Hydroxy-2-(2-(4-methylpiperidin-1-yl)-6-(trifluoromethyl)pyridin-3-yl)acetimidamide* (**13**). Compound **13** was synthesized from **12** according to general procedure C. 94% yield, white solid; ^1^H NMR (400 MHz, DMSO-*d*_6_) *δ* 9.01 (s, 1H), 7.69 (d, *J* = 7.8 Hz, 1H), 7.36 (d, *J* = 7.8 Hz, 1H), 5.53 (s, 2H), 3.38 (d, *J* = 12.8 Hz, 2H), 3.32 (s, 2H), 2.68 (t, *J* = 11.4 Hz, 2H), 1.66 (d, *J* = 12.8 Hz, 2H), 1.45–1.54 (m, 1H), 1.25 (qd, *J* = 12.0, 3.4 Hz, 2H), 0.92 (d, *J* = 6.4 Hz, 3H).
*Methyl 2-cyano-3-(2-(4-methylpiperidin-1-yl)-6-(trifluoromethyl)pyridin-3-yl)propanoate* (**14**). Compound **14** was synthesized from **11** according to general procedure D. 76% yield, pale yellow oil; ^1^H NMR (400 MHz, CDCl_3_) *δ* 7.62 (d, *J* = 7.8 Hz, 1H), 7.28 (d, *J* = 7.8 Hz, 1H), 4.33 (dd, *J* = 9.1, 5.9 Hz, 1H), 4.25 (qd, *J* = 7.2, 1.4 Hz, 2H), 3.45 (dd, *J* = 14.6, 5.9 Hz, 1H), 3.32 (t, *J* = 11.0 Hz, 2H), 3.13 (dd, *J* = 14.4, 9.4 Hz, 1H), 2.95 (td, *J* = 12.5, 2.4 Hz, 1H), 2.83 (td, *J* = 12.3, 2.3 Hz, 1H), 1.77 (t, *J* = 12.2 Hz, 2H), 1.54–1.61 (m, 1H), 1.31–1.40 (m, 2H), 0.99 (d, *J* = 6.4 Hz, 3H).
*3-(2-(4-Methylpiperidin-1-yl)-6-(trifluoromethyl)pyridin-3-yl)propanenitrile* (**15**). Compound **15** was synthesized from **14** according to general procedure E. 38% yield, pale yellow oil; ^1^H NMR (400 MHz, CDCl_3_) *δ* 7.57 (d, *J* = 7.8 Hz, 1H), 7.26 (d, *J* = 7.8 Hz, 1H), 3.32 (d, *J* = 12.9 Hz, 2H), 3.02 (t, *J* = 7.6 Hz, 2H), 2.87 (td, *J* = 12.6, 2.1 Hz, 2H), 2.74 (t, *J* = 7.4 Hz, 2H), 1.75 (d, *J* = 12.9 Hz, 2H), 1.55–1.61 (m, 1H), 1.32 (qd, *J* = 12.2, 3.9 Hz, 2H), 0.99 (d, *J* = 6.9 Hz, 3H).
*N’-Hydroxy-3-(2-(4-methylpiperidin-1-yl)-6-(trifluoromethyl)pyridin-3-yl)propanimidamide* (**16**). Compound **16** was synthesized from **15** according to general procedure C. 85% yield, colorless oil; ^1^H NMR (400 MHz, DMSO-*d*_6_) *δ* 8.80 (s, 1H), 7.75 (d, *J* = 7.8 Hz, 1H), 7.35 (d, *J* = 7.8 Hz, 1H), 5.41 (s, 2H), 3.34 (d, *J* = 12.9 Hz, 2H), 2.82 (t, *J* = 7.8 Hz, 2H), 2.70 (t, *J* = 11.3 Hz, 2H), 2.30 (t, *J* = 8.0 Hz, 2H), 1.67 (d, *J* = 10.6 Hz, 2H), 1.45–1.53 (m, 1H), 1.24 (qd, *J* = 12.2, 3.4 Hz, 2H), 0.92 (d, *J* = 6.4 Hz, 3H).
*4-(3-Chlorophenyl)-2-(trifluoromethyl)thiazole-5-carboxamide* (**19**). **17** (1.0 equiv.) was dissolved in ammonia solution 7 N in methanol (40 equiv.) and the mixture was heated at 60 °C for 15 h. After completion of the reaction, the solvent was concentrated in vacuo. The residue was purified by column chromatography on silica gel to afford the desired compound. 94% yield, white solid; ^1^H NMR (400 MHz, CDCl_3_) *δ* 7.70 (t, *J* = 1.8 Hz, 1H), 7.57 (dt, *J* = 7.3, 1.6 Hz, 1H), 7.44–7.51 (m, 2H), 5.75 (s, 2H).
*4-(3-Chlorophenyl)-2-(trifluoromethyl)thiazole-5-carbonitrile* (**20**). Pyridine (30 equiv.) was added to a solution of **19** (1.0 equiv.) in THF at room temperature. After 30 min, trifluoroacetic anhydride (1.2 equiv.) was then added slowly at 0 °C, and the mixture was allowed to stir for an additional 1 h at room temperature. The reaction mixture was quenched with water and extracted with EtOAc. The organic layer was dried over MgSO_4_ and concentrated in vacuo. The residue was purified by column chromatography on silica gel to afford the desired compound. 91% yield, white solid; ^1^H NMR (400 MHz, CDCl_3_) *δ* 8.14 (t, *J* = 1.8 Hz, 1H), 8.06 (dt, *J* = 7.3, 1.6 Hz, 1H), 7.46–7.52 (m, 2H).
*4-(3-Chlorophenyl)-N’-hydroxy-2-(trifluoromethyl)thiazole-5-carboximidamide* (**21**). Compound **21** was synthesized from **20** according to general procedure C. 57% yield, white solid; ^1^H NMR (400 MHz, DMSO-*d*_6_) *δ* 10.15 (s, 1H), 7.74–7.75 (m, 1H), 7.68–7.71 (m, 1H), 7.48–7.49 (m, 2H), 6.17 (s, 2H).
*(4-(3-Chlorophenyl)-2-(trifluoromethyl)thiazol-5-yl)methanol* (**22**). Compound **22** was synthesized from **17** according to general procedure A. 80% yield, white solid; ^1^H NMR (400 MHz, CDCl_3_) *δ* 7.66–7.67 (m, 1H), 7.51–7.54 (m, 1H), 7.39–7.40 (m, 2H), 5.02 (s, 2H).
*(2-(tert-Butyl)-4-(3-chlorophenyl)thiazol-5-yl)methanol* (**23**). Compound **23** was synthesized from **18** according to general procedure A. 95% yield, yellow oil; ^1^H NMR (400 MHz, CDCl_3_) *δ* 7.71–7.72 (m, 1H), 7.56 (dt, *J* = 7.2, 1.6 Hz, 1H), 7.32–7.39 (m, 2H), 4.88 (s, 2H), 1.47 (s, 9H).
*2-(4-(3-Chlorophenyl)-2-(trifluoromethyl)thiazol-5-yl)acetonitrile* (**24**). Compound **24** was synthesized from **22** according to general procedure B. 38% yield, pale yellow oil; ^1^H NMR (400 MHz, CDCl_3_) *δ* 7.59–7.61 (m, 1H), 7.44–7.46 (m, 3H), 4.04 (s, 2H).
*2-(2-(tert-Butyl)-4-(3-chlorophenyl)thiazol-5-yl)acetonitrile* (**25**). Compound **25** was synthesized from **23** according to general procedure B. 77% yield, yellow oil; ^1^H NMR (400 MHz, CDCl_3_) *δ* 7.57–7.58 (m, 1H), 7.35–7.43 (m, 3H), 3.91 (s, 2H), 1.45 (s, 9H).
*2-(4-(3-Chlorophenyl)-2-(trifluoromethyl)thiazol-5-yl)-N’-hydroxyacetimidamide* (**26**). Compound **26** was synthesized from **24** according to general procedure C. 64% yield, pale yellow oil; ^1^H NMR (400 MHz, DMSO-*d*_6_) *δ* 9.23 (s, 1H), 7.68–7.69 (m, 1H), 7.61–7.64 (m, 1H), 7.50–7.52 (m, 2H), 5.74 (s, 2H), 3.76 (s, 2H).
*2-(2-(tert-Butyl)-4-(3-chlorophenyl)thiazol-5-yl)-N’-hydroxyacetimidamide* (**27**). Compound **27** was synthesized from **25** according to general procedure C. 91% yield, colorless oil; ^1^H NMR (400 MHz, DMSO-*d*_6_) *δ* 9.10 (s, 1H), 7.67 (t, *J* = 1.8 Hz, 1H), 7.61 (dt, *J* = 7.4, 1.4 Hz, 1H), 7.38–7.47 (m, 2H), 5.59 (s, 2H), 3.56 (s, 2H), 1.35 (s, 9H).
*Methyl 3-(4-(3-chlorophenyl)-2-(trifluoromethyl)thiazol-5-yl)-2-cyanopropanoate* (**28**). Compound **28** was synthesized from **22** according to general procedure D. 95% yield, colorless oil; ^1^H NMR (400 MHz, CDCl_3_) *δ* 7.58–7.59 (m, 1H), 7.42–7.48 (m, 3H), 3.83 (s, 3H), 3.76 (dd, *J* = 7.5, 5.7 Hz, 1H), 3.69 (dd, *J* = 15.3, 5.9 Hz, 1H), 3.60 (q, *J* = 7.6 Hz, 1H).
*3-(4-(3-Chlorophenyl)-2-(trifluoromethyl)thiazol-5-yl)propanenitrile* (**29**). Compound **29** was synthesized from **28** according to general procedure E. 54% yield, yellow oil; ^1^H NMR (400 MHz, CDCl_3_) *δ* 7.57–7.58 (m, 1H), 7.41–7.47 (m, 3H), 3.37 (t, *J* = 7.1 Hz, 2H), 2.70 (t, *J* = 7.4 Hz, 2H).
*3-(4-(3-Chlorophenyl)-2-(trifluoromethyl)thiazol-5-yl)-N’-hydroxypropanimidamide* (**30**). Compound **30** was synthesized from **29** according to general procedure C. 77% yield, colorless oil; ^1^H NMR (400 MHz, DMSO-*d*_6_) *δ* 8.93 (s, 1H), 7.62–7.63 (m, 1H), 7.56–7.58 (m, 1H), 7.50–7.51 (m, 2H), 5.48 (s, 2H), 3.21 (t, *J* = 7.1 Hz, 2H), 2.36 (t, *J* = 7.4 Hz, 2H).
*Ethyl 3-(tert-butyl)-1-(3-chlorophenyl)-1H-pyrazole-5-carboxylate* (**32**). To a solution of **31** (1.0 equiv.) in CH_2_Cl_2_ were added (3-chlorophenyl)boronic acid (2.0 equiv.), copper(II) acetate (1.5 equiv.), and pyridine (2.0 equiv.). The mixture was stirred for 22 h at room temperature, filtered through Celite, and the filtrate was concentrated in vacuo. The residue was purified by column chromatography on silica gel to afford the desired compound. 84% yield, yellow oil; ^1^H NMR (400 MHz, CDCl_3_) *δ* 7.44–7.45 (m, 1H), 7.30–7.37 (m, 3H), 6.88 (s, 1H), 4.23 (q, *J* = 7.2 Hz, 2H), 1.34 (s, 9H), 1.25 (t, *J* = 7.1 Hz, 3H).
*2-(3-(tert-Butyl)-1-(3-chlorophenyl)-1H-pyrazol-5-yl)acetonitrile* (**33**). Compound **33** was synthesized from **32** according to general procedures A and B. 84% yield, pale yellow oil; ^1^H NMR (400 MHz, CDCl_3_) *δ* 7.38–7.45 (m, 3H), 7.29 (dt, *J* = 7.3, 1.8 Hz, 1H), 6.41 (s, 1H), 3.73 (s, 2H), and 1.33 (s, 9H).
*2-(3-(tert-Butyl)-1-(3-chlorophenyl)-1H-pyrazol-5-yl)-N’-hydroxyacetimidamide* (**34**). Compound **34** was synthesized from **33** according to general procedure C. 95% yield, white solid; ^1^H NMR (400 MHz, DMSO-*d*_6_) *δ* 9.02 (s, 1H), 7.59–7.60 (m, 1H), 7.40–7.53 (m, 3H), 6.24 (s, 1H), 5.51 (s, 2H), 3.36 (s, 2H), 1.22 (s, 9H).
*Methyl 2-oxo-2,3-dihydro-1H-benzo[d]imidazole-4-carboxylate* (**36**). To a solution of methyl 2,3-diaminobenzoate (1.0 equiv.) in THF were added 1,1′-carbonyldiimidazole (1.05 equiv.) and triethylamine (1.0 equiv.) and the mixture was refluxed for 21 h. After completion of the reaction, the solvent was concentrated in vacuo. After recrystallization with hexane, the desired compound was isolated. 95% yield, light brown solid; ^1^H NMR (400 MHz, DMSO-*d*_6_) *δ* 10.93 (s, 1H), 10.68 (s, 1H), 7.44 (dd, *J* = 8.0, 1.1 Hz, 1H), 7.13 (d, *J* = 7.4 Hz, 1H), 7.01 (t, *J* = 7.8 Hz, 1H), 3.82 (s, 3H).
*2-Oxo-2,3-dihydro-1H-benzo[d]imidazole-4-carboxylic acid* (**37**). Compound **37** was synthesized from **36** according to general procedure F. 75% yield, brown solid; ^1^H NMR (400 MHz, DMSO-*d*_6_) *δ* 10.85 (s, 1H), 10.36 (s, 1H), 7.40 (dd, *J* = 8.0, 1.1 Hz, 1H), 7.09 (d, *J* = 7.4 Hz, 1H), and 6.97 (t, *J* = 7.8 Hz, 1H).
*Methyl 2-(3-fluoro-2-nitrophenyl)acetate* (**39**). Dimethylmalonate (1.5 equiv.) was added slowly to a suspension of sodium hydride with 60% dispersion in paraffin liquid (1.5 equiv.) in DMF at 0 °C, and the mixture was stirred for 30 min at room temperature. 1,3-difluoro-2-nitrobenzene (1.0 equiv.) was then added slowly at 0 °C, and the mixture was allowed to stir at 50 °C for 15 h. After dilution with water at 0 °C, the reaction mixture was quenched with 1 N HCl and extracted with EtOAc. The organic layer was dried over MgSO_4_, and concentrated in vacuo. The residue was purified by column chromatography on silica gel to afford the desired compound. 90% yield, pale yellow solid; ^1^H NMR (400 MHz, CDCl_3_) *δ* 7.47 (td, *J* = 8.3, 5.5 Hz, 1H), 7.21 (t, *J* = 8.9 Hz, 1H), 7.15 (d, *J* = 7.3 Hz, 1H), 3.82 (s, 2H), and 3.70 (s, 3H).
*Methyl 2-(3-(benzylamino)-2-nitrophenyl)acetate* (**40**). Methyl 2-(3-fluoro-2-nitrophenyl)acetate (1.0 equiv.), synthesized from **39** according to general procedure, was added to a solution of benzylamine (1.5 equiv.) in DMF. E. Triethylamine (1.5 equiv.) was then added, and the mixture was stirred at 60 °C for 13 h. The reaction mixture was quenched with water and extracted with EtOAc. The organic layer was dried over MgSO_4_, and concentrated in vacuo. The residue was purified by column chromatography on silica gel to afford the desired compound. 89% yield, orange oil; ^1^H NMR (400 MHz, CDCl_3_) *δ* 7.57 (s, 1H), 7.23–7.37 (m, 6H), 6.76 (d, *J* = 8.6 Hz, 1H), 6.53 (d, *J* = 7.3 Hz, 1H), 4.47 (d, *J* = 5.5 Hz, 2H), 3.88 (s, 2H), and 3.71 (s, 3H).
*2-(2-Oxo-2,3-dihydro-1H-benzo[d]imidazol-4-yl)acetic acid* (**41**). Palladium 10% on activated carbon was added to a solution of **40** (1.0 equiv.) in methanol and the mixture was stirred under hydrogen for 1 h at room temperature. The mixture was filtered through Celite and the filterate was concentrated in vacuo. 1,1′-carbonyldiimidazole (1.1 equiv.) was added to a solution of obtained residue in THF and the mixture was stirred for 1 h at room temperature. The reaction mixture was quenched with water and extracted with EtOAc. The organic layer was dried over MgSO_4_, and concentrated in vacuo. The residue was purified by column chromatography on silica gel. Compound **41** was synthesized from the obtained residue according to general procedure F. 86% yield, light brown solid; ^1^H NMR (400 MHz, DMSO-*d*_6_) *δ* 10.64 (s, 1H), 10.54 (s, 1H), and 6.73–6.84 (m, 3H), 3.58 (s, 2H).
*7-Methyl-1,7-dihydro-6H-purin-6-one* (**43**). Under a nitrogen atmosphere, methylmagnesium chloride 3.0 M solution in THF (1.1 equiv.) was slowly added to a solution of 6-chloro-9H-purine (1.0 equiv.) in anhydrous THF, and the mixture was stirred for 1 h at room temperature. Iodomethane (3.0 equiv.) was then added, and the mixture was allowed to stir at 50 °C for 15 h. The reaction mixture was quenched with water and extracted with EtOAc. The organic layer was dried over MgSO_4_, and concentrated in vacuo. The residue was purified by column chromatography on silica gel. The obtained residue was dissolved in formic acid and heated to 75 °C. After concentration in vacuo, the residue was re-dissolved in ethanol, and the mixture was refluxed for an additional 30 min. The solvent was concentrated in vacuo to afford the desired compound, which was used in the next step without further purification. 86% yield, pale yellow solid; ^1^H NMR (400 MHz, DMSO-*d*_6_) *δ* 12.36 (s, 1H), 8.27 (s, 1H), 7.95 (s, 1H), and 3.93 (s, 3H).
*tert-Butyl 2-(7-methyl-6-oxo-6,7-dihydro-1H-purin-1-yl)acetate* (**44**). To a stirred solution of **43** (1.0 equiv.), tert-butyl bromoacetate (1.0 equiv.) and potassium carbonate (1.2 equiv.) in DMF was added, and the mixture was stirred at 50 °C for 6 h. The reaction mixture was quenched with water and extracted with EtOAc. The organic layer was dried over MgSO_4_ and concentrated in vacuo. The residue was purified by column chromatography on silica gel to afford the desired compound. 36% yield, white solid; ^1^H NMR (400 MHz, DMSO-*d*_6_) *δ* 8.21 (s, 1H), 8.16 (s, 1H), 4.67 (s, 2H), 3.92 (s, 3H), and 1.39 (s, 9H).
*2-(7-Methyl-6-oxo-6,7-dihydro-1H-purin-1-yl)acetic acid* (**45**). **44** was dissolved in hydrochloric acid and the mixture was stirred for 30 min at room temperature. After completion of the reaction, the solvent was concentrated in vacuo to afford the desired compound, which was used in the next step without further purification. 99% yield, white solid; ^1^H NMR (400 MHz, DMSO-*d*_6_) *δ* 8.27 (s, 1H), 8.25 (s, 1H), 4.70 (s, 2H), 3.93 (s, 3H).
*4-(3-(4-Chlorophenethyl)-1,2,4-oxadiazol-5-yl)-1,3-dihydro-2H-benzo[d]imidazol-2-one* (**46**). Compound **46** was synthesized from 3-(4-chlorophenyl)-N’-hydroxypropanimidamide and **37** according to general procedure G. 72% yield, 99.01% purity, white solid; ^1^H NMR (400 MHz, DMSO-*d*_6_) *δ* 11.09 (s, 1H), 10.74 (s, 1H), 7.55 (dd, *J* = 8.0, 1.1 Hz, 1H), 7.26–7.31 (m, 4H), 7.18 (dd, *J* = 7.8, 0.9 Hz, 1H), 7.10 (t, *J* = 8.0 Hz, 1H), 3.11–3.14 (m, 2H), 3.02–3.06 (m, 2H); ^13^C NMR (100 MHz, DMSO-*d*_6_) *δ* 173.39, 170.36, 155.56, 140.06, 131.41, 131.31, 130.88, 129.24, 128.79, 121.63, 119.73, 113.06, 104.91, 31.68, 27.42; HRMS (FAB) calc. for C_17_H_13_ClN_4_O_2_ [M + H]^+^ 341.0805, found: 341.0795.
*4-(3-((4′-(Trifluoromethyl)-[1,1′-biphenyl]-3-yl)methyl)-1,2,4-oxadiazol-5-yl)-1,3-dihydro-2H-benzo[d]imidazol-2-one* (**47**). Compound **47** was synthesized from N’-hydroxy-2-(4′-(trifluoromethyl)-[1,1′-biphenyl]-3-yl)acetimidamide and **37** according to general procedure G. 55% yield, 99.47% purity, white solid; ^1^H NMR (400 MHz, DMSO-*d*_6_) *δ* 11.09 (s, 1H), 10.74 (s, 1H), 7.86 (d, *J* = 8.2 Hz, 2H), 7.77–7.79 (m, 3H), 7.60 (td, *J* = 4.3, 2.3 Hz, 1H), 7.55 (dd, *J* = 8.0, 1.1 Hz, 1H), 7.43–7.48 (m, 2H), 7.16 (d, *J* = 7.3 Hz, 1H), 7.09 (t, *J* = 7.8 Hz, 1H), 4.24 (s, 2H); ^13^C NMR (100 MHz, DMSO-*d*_6_) *δ* 173.87, 170.07, 155.55, 144.47, 139.39, 137.33, 131.42, 129.92, 129.71, 129.26, 128.43 (d, *J* = 31.5 Hz), 128.42, 128.08, 126.35, 126.31, 126.27, 126.22, 121.64, 119.89, 113.11, 104.89, 31.95; HRMS (FAB) calc. for C_23_H_15_F_3_N_4_O_2_ [M + H]^+^ 437.1225, found: 437.1241.
*4-(3-((4′-(Trifluoromethyl)-[1,1′-biphenyl]-4-yl)methyl)-1,2,4-oxadiazol-5-yl)-1,3-dihydro-2H-benzo[d]imidazol-2-one* (**48**). Compound **48** was synthesized from N’-hydroxy-2-(4′-(trifluoromethyl)-[1,1′-biphenyl]-4-yl)acetimidamide and **37** according to general procedure G. 71% yield, 98.85% purity, white solid; ^1^H NMR (400 MHz, DMSO-*d*_6_) *δ* 11.10 (s, 1H), 10.75 (s, 1H), 7.85 (d, *J* = 8.2 Hz, 2H), 7.76 (d, *J* = 8.2 Hz, 2H), 7.69 (d, *J* = 8.2 Hz, 2H), 7.52–7.57 (m, 3H), 7.17 (d, *J* = 6.9 Hz, 1H), 7.10 (t, *J* = 7.8 Hz, 1H), 4.22 (s, 2H); ^13^C NMR (100 MHz, DMSO-*d*_6_) *δ* 173.92, 170.03, 155.57, 144.36, 137.76, 136.79, 131.41, 130.38, 129.23, 128.29 (d, *J* = 31.6 Hz), 127.92, 127.79, 126.32, 126.30, 126.26, 121.66, 119.93, 113.13, 104.86, 31.60; HRMS (FAB) calc. for C_23_H_15_F_3_N_4_O_2_ [M + H]^+^ 437.1225, found: 437.1208.
*4-(3-(2-(4-Methylpiperidin-1-yl)-6-(trifluoromethyl)pyridin-3-yl)-1,2,4-oxadiazol-5-yl)-1,3-dihydro-2H-benzo[d]imidazol-2-one* (**49**). Compound **49** was synthesized from **10** and **37** according to general procedure G. 59% yield, 99.35% purity, pale yellow solid; ^1^H NMR (400 MHz, DMSO-*d*_6_) *δ* 11.13 (s, 1H), 10.96 (s, 1H), 8.79 (d, *J* = 7.3 Hz, 1H), 7.65 (dd, *J* = 8.0, 1.1 Hz, 1H), 7.36 (d, *J* = 7.8 Hz, 1H), 7.21 (d, *J* = 7.3 Hz, 1H), 7.15 (t, *J* = 7.8 Hz, 1H), 3.66 (d, *J* = 13.3 Hz, 2H), 2.81 (t, *J* = 11.7 Hz, 2H), 1.59 (d, *J* = 12.3 Hz, 2H), 1.48–1.53 (m, 1H), 1.23 (qd, *J* = 12.1, 3.4 Hz, 2H), 0.88 (d, *J* = 5.9 Hz, 3H); ^13^C NMR (100 MHz, DMSO-*d*_6_) *δ* 173.32, 167.48, 159.33, 155.65, 144.14, 131.48, 129.39, 121.82 (d, *J* = 275.9 Hz), 121.68, 119.89, 114.22, 113.32, 111.34, 104.61, 49.66, 33.86, 30.63, 22.32; HRMS (FAB) calc. for C_21_H_19_F_3_N_6_O_2_ [M + H]^+^ 445.1600, found: 445.1592.
*4-(3-((2-(4-Methylpiperidin-1-yl)-6-(trifluoromethyl)pyridin-3-yl)methyl)-1,2,4-oxadiazol-5-yl)-1,3-dihydro-2H-benzo[d]imidazol-2-one* (**50**). Compound **50** was synthesized from **13** and **37** according to general procedure G. 30% yield, 99.42% purity, brown solid; ^1^H NMR (400 MHz, DMSO-*d*_6_) *δ* 11.10 (s, 1H), 10.75 (s, 1H), 7.89 (d, *J* = 7.8 Hz, 1H), 7.55 (dd, *J* = 8.2, 0.9 Hz, 1H), 7.41 (d, *J* = 7.8 Hz, 1H), 7.18 (d, *J* = 7.8 Hz, 1H), 7.10 (t, *J* = 8.0 Hz, 1H), 4.23 (s, 2H), 3.39 (d, *J* = 12.8 Hz, 2H), 2.74 (t, *J* = 11.4 Hz, 2H), 1.66 (d, *J* = 12.8 Hz, 2H), 1.46–1.51 (m, 1H), 1.20–1.29 (m, 2H), 0.90 (d, *J* = 6.4 Hz, 3H); ^13^C NMR (100 MHz, DMSO-*d*_6_) *δ* 173.97, 169.32, 162.34, 155.58, 143.80, 141.25, 131.42, 129.23, 128.00, 122.12 (d, *J* = 272.2 Hz), 121.67, 119.98, 114.77, 113.18, 104.82, 50.75, 34.31, 30.69, 27.88, 22.38; HRMS (FAB) calc. for C_22_H_21_F_3_N_6_O_2_ [M + H]^+^ 459.1756, found: 459.1762.
*4-(3-(2-(2-(4-Methylpiperidin-1-yl)-6-(trifluoromethyl)pyridin-3-yl)ethyl)-1,2,4-oxadiazol-5-yl)-1,3-dihydro-2H-benzo[d]imidazol-2-one* (**51**). Compound **51** was synthesized from **16** and **37** according to general procedure G. 57% yield, 99.45% purity, pale yellow solid; ^1^H NMR (400 MHz, DMSO-*d*_6_) *δ* 11.09 (s, 1H), 10.66 (s, 1H), 7.85 (d, *J* = 7.8 Hz, 1H), 7.56 (dd, *J* = 8.0, 1.1 Hz, 1H), 7.37 (d, *J* = 7.4 Hz, 1H), 7.18 (dd, *J* = 7.8, 0.9 Hz, 1H), 7.11 (t, *J* = 7.8 Hz, 1H), 3.34–3.36 (m, 2H), 3.13–3.19 (m, 4H), 2.71 (t, *J* = 11.4 Hz, 2H), 1.64 (d, *J* = 11.0 Hz, 2H), 1.43–1.48 (m, 1H), 1.19–1.26 (m, 2H), 0.86 (d, *J* = 6.4 Hz, 3H); ^13^C NMR (100 MHz, DMSO-*d*_6_) *δ* 173.50, 170.42, 162.50, 155.50, 142.99 (q, *J* = 33.4 Hz), 140.01, 132.27, 131.41, 129.25, 122.22 (d, *J* = 271.1 Hz), 121.65, 119.74, 114.80, 113.08, 104.92, 50.86, 34.37, 30.76, 28.36, 25.49, 22.25; HRMS (FAB) calc. for C_23_H_23_F_3_N_6_O_2_ [M + H]^+^ 473.1913, found: 473.1906.
*4-(3-(4-(3-Chlorophenyl)-2-(trifluoromethyl)thiazol-5-yl)-1,2,4-oxadiazol-5-yl)-1,3-dihydro-2H-benzo[d]imidazol-2-one* (**52**). Compound **52** was synthesized from **21** and **37** according to general procedure G. 64% yield, 99.28% purity, pale yellow solid; ^1^H NMR (400 MHz, DMSO-*d*_6_) *δ* 11.14 (s, 1H), 10.75 (s, 1H), 7.89 (t, *J* = 1.8 Hz, 1H), 7.76 (dt, *J* = 7.3, 1.4 Hz, 1H), 7.55–7.58 (m, 2H), 7.51 (t, *J* = 8.0 Hz, 1H), 7.21 (dd, *J* = 7.8, 0.9 Hz, 1H), 7.12 (t, *J* = 8.0 Hz, 1H); ^13^C NMR (100 MHz, DMSO-*d*_6_) *δ* 174.21, 162.07, 155.77 (d, *J* = 40.1 Hz), 155.54, 154.85, 134.78, 133.44, 131.52, 130.73, 130.31, 129.84, 129.48, 128.88, 124.19, 121.76, 120.10, 119.90 (d, *J* = 270.2 Hz), 113.67, 103.99; HRMS (FAB) calc. for C_19_H_9_ClF_3_N_5_O_2_S [M + H]^+^ 464.0196, found: 464.0211.
*4-(3-(2-(4-(3-Chlorophenyl)-2-(trifluoromethyl)thiazol-5-yl)ethyl)-1,2,4-oxadiazol-5-yl)-1,3-dihydro-2H-benzo[d]imidazol-2-one* (**53**). Compound **53** was synthesized from **30** and **37** according to general procedure G. 37% yield, 98.60% purity, pale yellow solid; ^1^H NMR (400 MHz, DMSO-*d*_6_) *δ* 11.09 (s, 1H), 10.66 (s, 1H), 7.57–7.59 (m, 2H), 7.50 (d, *J* = 6.9 Hz, 1H), 7.44 (t, *J* = 7.8 Hz, 1H), 7.37–7.40 (m, 1H), 7.18 (dd, *J* = 7.5, 1.1 Hz, 1H), 7.10 (t, *J* = 8.0 Hz, 1H), 3.63 (t, *J* = 7.1 Hz, 2H), 3.17 (t, *J* = 7.1 Hz, 2H); ^13^C NMR (100 MHz, DMSO-*d*_6_) *δ* 173.66, 169.42, 155.54, 151.57, 150.96 (d, *J* = 40.1 Hz), 139.59, 135.49, 133.93, 131.38, 131.09, 129.31, 129.10, 128.76, 127.81, 121.59, 119.72, 118.89, 113.14, 104.71, 27.50, 24.14; HRMS (FAB) calc. for C_21_H_13_ClF_3_N_5_O_2_S [M + H]^+^ 492.0509, found: 492.0519.
*4-(3-((2-(tert-Butyl)-4-(3-chlorophenyl)thiazol-5-yl)methyl)-1,2,4-oxadiazol-5-yl)-1,3-dihydro-2H-benzo[d]imidazol-2-one* (**54**). Compound **54** was synthesized from **27** and **37** according to general procedure G. 43% yield, 98.83% purity, pale yellow solid; ^1^H NMR (400 MHz, DMSO-*d*_6_) *δ* 11.10 (s, 1H), 10.70 (s, 1H), 7.74 (s, 1H), 7.66 (d, *J* = 7.8 Hz, 1H), 7.57 (dd, *J* = 8.0, 1.1 Hz, 1H), 7.43–7.51 (m, 2H), 7.18 (d, *J* = 7.8 Hz, 1H), 7.11 (t, *J* = 8.0 Hz, 1H), 4.47 (s, 2H), 1.35 (s, 9H); ^13^C NMR (100 MHz, DMSO-*d*_6_) *δ* 179.07, 174.29, 169.32, 155.52, 149.84, 136.84, 133.82, 131.47, 131.03, 129.34, 128.75, 128.43, 127.63, 127.18, 121.71, 120.05, 113.29, 104.67, 37.92, 30.98, 24.41; HRMS (FAB) calc. for C_23_H_20_ClN_5_O_2_S [M + H]^+^ 466.1104, found: 466.1100.
*4-(3-((3-(tert-Butyl)-1-(3-chlorophenyl)-1H-pyrazol-5-yl)methyl)-1,2,4-oxadiazol-5-yl)-1,3-dihydro-2H-benzo[d]imidazol-2-one* (**55**). Compound **55** was synthesized from **34** and **37** according to general procedure G. 33% yield, 99.30% purity, light brown solid; ^1^H NMR (400 MHz, DMSO-*d*_6_) *δ* 11.08 (s, 1H), 10.62 (s, 1H), 7.64 (t, *J* = 2.1 Hz, 1H), 7.46–7.54 (m, 3H), 7.43 (dt, *J* = 7.8, 1.8 Hz, 1H), 7.17 (dd, *J* = 7.8, 0.9 Hz, 1H), 7.10 (t, *J* = 8.0 Hz, 1H), 6.32 (s, 1H), 4.33 (s, 2H), 1.22 (s, 9H); ^13^C NMR (100 MHz, DMSO-*d*_6_) *δ* 174.00, 168.20, 162.29, 155.49, 141.18, 138.05, 133.97, 131.42, 131.36, 129.27, 127.94, 125.02, 123.75, 121.66, 119.93, 113.19, 105.51, 104.74, 32.41, 30.77, 24.04; HRMS (FAB) calc. for C_23_H_21_ClN_6_O_2_ [M + H]^+^ 449.1493, found: 449.1487.
*4-((3-(4-Chlorophenethyl)-1,2,4-oxadiazol-5-yl)methyl)-1,3-dihydro-2H-benzo[d]imidazol-2-one* (**56**). Compound **56** was synthesized from 3-(4-chlorophenyl)-N’-hydroxypropanimidamide and **41** according to general procedure G. 38% yield, 97.77% purity, pale yellow solid; ^1^H NMR (400 MHz, DMSO-*d*_6_) *δ* 10.82 (s, 1H), 10.68 (s, 1H), 7.24–7.27 (m, 2H), 7.17–7.20 (m, 2H), 6.82–6.89 (m, 2H), 6.77 (dd, *J* = 7.3, 1.4 Hz, 1H), 4.31 (s, 2H), 2.89–2.93 (m, 4H); ^13^C NMR (100 MHz, DMSO-*d*_6_) *δ* 178.07, 169.91, 155.79, 139.73, 131.31, 130.84, 130.38, 129.32, 128.72, 121.97, 121.19, 115.08, 108.23, 31.84, 27.86, 27.30; HRMS (FAB) calc. for C_18_H_15_ClN_4_O_2_ [M + H]^+^ 355.0962, found: 355.0951.
*4-((3-((4′-(Trifluoromethyl)-[1,1′-biphenyl]-3-yl)methyl)-1,2,4-oxadiazol-5-yl)methyl)-1,3-dihydro-2H-benzo[d]imidazol-2-one* (**57**). Compound **57** was synthesized from N’-hydroxy-2-(4′-(trifluoromethyl)-[1,1′-biphenyl]-3-yl)acetimidamide and **41** according to general procedure G. 38% yield, 99.26% purity, pale yellow solid; ^1^H NMR (400 MHz, DMSO-*d*_6_) *δ* 10.81 (s, 1H), 10.67 (s, 1H), 7.77–7.81 (m, 4H), 7.57–7.61 (m, 2H), 7.42 (t, *J* = 7.8 Hz, 1H), 7.31 (d, *J* = 7.8 Hz, 1H), 6.78–6.87 (m, 3H), 4.31 (s, 2H), 4.11 (s, 2H); ^13^C NMR (100 MHz, DMSO-*d*_6_) *δ* 178.46, 169.71, 155.77, 144.45, 139.39, 137.26, 130.37, 129.89, 129.56, 129.39, 128.44 (d, *J* = 32.5 Hz), 128.21, 128.03, 126.37, 126.33, 126.25, 126.19, 122.07, 121.16, 114.95, 108.25, 31.74, 27.98; HRMS (FAB) calc. for C_24_H_17_F_3_N_4_O_2_ [M + H]^+^ 451.1382, found: 451.1393.
*4-((3-((4′-(Trifluoromethyl)-[1,1′-biphenyl]-4-yl)methyl)-1,2,4-oxadiazol-5-yl)methyl)-1,3-dihydro-2H-benzo[d]imidazol-2-one* (**58**). Compound **58** was synthesized from N’-hydroxy-2-(4′-(trifluoromethyl)-[1,1′-biphenyl]-4-yl)acetimidamide and **41** according to general procedure G. 56% yield, 98.98% purity, white solid; ^1^H NMR (400 MHz, DMSO-*d*_6_) *δ* 10.81 (s, 1H), 10.67 (s, 1H), 7.83 (d, *J* = 8.2 Hz, 2H), 7.76 (d, *J* = 8.2 Hz, 2H), 7.65 (d, *J* = 8.2 Hz, 2H), 7.38 (d, *J* = 8.2 Hz, 2H), 6.78–6.88 (m, 3H), 4.32 (s, 2H), 4.08 (s, 2H); ^13^C NMR (100 MHz, DMSO-*d*_6_) *δ* 178.43, 169.68, 155.77, 144.34, 137.73, 136.69, 130.37, 130.25, 129.38, 128.31 (d, *J* = 31.5 Hz), 127.92, 127.76, 126.32, 126.28, 126.25, 122.07, 121.19, 114.91, 108.27, 31.44, 27.99; HRMS (FAB) calc. for C_24_H_17_F_3_N_4_O_2_ [M + H]^+^ 451.1382, found: 451.1393.
*4-((3-(2-(4-Methylpiperidin-1-yl)-6-(trifluoromethyl)pyridin-3-yl)-1,2,4-oxadiazol-5-yl)methyl)-1,3-dihydro-2H-benzo[d]imidazol-2-one* (**59**). Compound **59** was synthesized from **10** and **41** according to general procedure G. 36% yield, 98.31% purity, yellow solid; ^1^H NMR (400 MHz, DMSO-*d*_6_) *δ* 10.84 (s, 1H), 10.69 (s, 1H), 8.06 (d, *J* = 7.8 Hz, 1H), 7.26 (d, *J* = 7.8 Hz, 1H), 6.83–6.91 (m, 3H), 4.43 (s, 2H), 3.51 (d, *J* = 13.3 Hz, 2H), 2.68 (t, *J* = 11.9 Hz, 2H), 1.38–1.45 (m, 3H), 0.92–1.01 (m, 2H), 0.81 (d, *J* = 5.9 Hz, 3H); ^13^C NMR (100 MHz, DMSO-*d*_6_) *δ* 178.80, 167.77, 158.87, 155.80, 146.65, 143.16, 130.43, 129.44, 122.21, 121.72 (d, *J* = 272.1 Hz), 121.18, 114.79, 113.80, 111.05, 108.34, 49.15, 33.61, 30.58, 28.05, 22.15; HRMS (FAB) calc. for C_22_H_21_F_3_N_6_O_2_ [M + H]^+^ 459.1756, found: 459.1749.
*4-((3-((2-(4-Methylpiperidin-1-yl)-6-(trifluoromethyl)pyridin-3-yl)methyl)-1,2,4-oxadiazol-5-yl)methyl)-1,3-dihydro-2H-benzo[d]imidazol-2-one* (**60**). Compound **60** was synthesized from **13** and **41** according to general procedure G. 73% yield, 98.85% purity, light brown solid; ^1^H NMR (400 MHz, DMSO-*d*_6_) *δ* 10.79 (s, 1H), 10.67 (s, 1H), 7.69 (d, *J* = 7.3 Hz, 1H), 7.38 (d, *J* = 7.8 Hz, 1H), 6.76–6.87 (m, 3H), 4.31 (s, 2H), 4.10 (s, 2H), 3.27–3.30 (m, 2H), 2.65 (t, *J* = 11.4 Hz, 1H), 1.56 (d, *J* = 12.8 Hz, 2H), 1.39–1.47 (m, 1H), 1.08 (qd, *J* = 12.0, 3.3 Hz, 2H), 0.84 (d, *J* = 6.4 Hz, 3H); ^13^C NMR (100 MHz, DMSO-*d*_6_) *δ* 178.39, 168.92, 162.31, 155.76, 143.64 (d, *J* = 33.6 Hz), 141.14, 130.35, 129.30, 128.00, 122.08 (d, *J* = 272.1 Hz), 121.96, 121.15, 114.90, 114.69, 108.26, 50.55, 34.15, 30.58, 27.93, 22.31; HRMS (FAB) calc. for C_23_H_23_F_3_N_6_O_2_ [M + H]^+^ 473.1913, found: 473.1916.
*4-((3-(2-(2-(4-Methylpiperidin-1-yl)-6-(trifluoromethyl)pyridin-3-yl)ethyl)-1,2,4-oxadiazol-5-yl)methyl)-1,3-dihydro-2H-benzo[d]imidazol-2-one* (**61**). Compound **61** was synthesized from **16** and **41** according to general procedure G. 68% yield, 99.58% purity, white solid; ^1^H NMR (400 MHz, DMSO-*d*_6_) *δ* 10.80 (s, 1H), 10.67 (s, 1H), 7.78 (d, *J* = 7.8 Hz, 1H), 7.32 (d, *J* = 7.8 Hz, 1H), 6.81–6.87 (m, 2H), 6.76 (dd, *J* = 7.1, 1.6 Hz, 1H), 4.30 (s, 2H), 3.29 (d, *J* = 12.8 Hz, 2H), 2.96–3.06 (m, 4H), 2.67 (t, *J* = 11.4 Hz, 2H), 1.62 (d, *J* = 10.5 Hz, 2H), 1.43–1.48 (m, 1H), 1.12–1.22 (m, 2H), 0.89 (d, *J* = 6.4 Hz, 3H); ^13^C NMR (100 MHz, DMSO-*d*_6_) *δ* 178.13, 170.07, 162.38, 155.78, 142.93 (d, *J* = 33.4 Hz), 139.77, 131.94, 130.38, 129.35, 122.20 (d, *J* = 271.1 Hz), 121.96, 121.12, 115.01, 114.65, 108.20, 50.78, 34.38, 30.76, 28.14, 27.86, 25.12, 22.34; HRMS (FAB) calc. for C_24_H_25_F_3_N_6_O_2_ [M + H]^+^ 487.2069, found: 487.2074.
*4-((3-(4-(3-Chlorophenyl)-2-(trifluoromethyl)thiazol-5-yl)-1,2,4-oxadiazol-5-yl)methyl)-1,3-dihydro-2H-benzo[d]imidazol-2-one* (**62**). Compound **62** was synthesized from **21** and **41** according to general procedure G. 31% yield, 99.61% purity, pale yellow solid; ^1^H NMR (400 MHz, DMSO-*d*_6_) *δ* 10.81 (s, 1H), 10.69 (s, 1H), 7.77 (t, *J* = 1.8 Hz, 1H), 7.65 (dt, *J* = 7.8, 1.4 Hz, 1H), 7.51–7.53 (m, 1H), 7.45 (t, *J* = 8.0 Hz, 1H), 6.83–6.90 (m, 3H), 4.43 (s, 2H); ^13^C NMR (100 MHz, DMSO-*d*_6_) *δ* 179.39, 161.76, 155.79, 155.41 (d, *J* = 39.3 Hz), 154.52, 134.49, 133.42, 130.68, 130.38, 130.20, 129.63, 129.38, 128.75, 123.72, 122.20, 121.23, 119.47 (d, *J* = 219.5 Hz), 114.25, 108.45, 28.00; HRMS (FAB) calc. for C_20_H_11_ClF_3_N_5_O_2_S [M + H]^+^ 478.0352, found: 478.0356.
*4-((3-((4-(3-Chlorophenyl)-2-(trifluoromethyl)thiazol-5-yl)methyl)-1,2,4-oxadiazol-5-yl)methyl)-1,3-dihydro-2H-benzo[d]imidazol-2-one* (**63**). Compound **63** was synthesized from **26** and **41** according to general procedure G. 31% yield, 99.42% purity, yellow solid; ^1^H NMR (400 MHz, DMSO-*d*_6_) *δ* 10.78 (s, 1H), 10.67 (s, 1H), 7.68 (s, 1H), 7.59 (dt, *J* = 6.7, 1.7 Hz, 1H), 7.46–7.52 (m, 2H), 6.82–6.88 (m, 2H), 6.78 (dd, *J* = 7.4, 1.4 Hz, 1H), 4.56 (s, 2H), 4.34 (s, 2H); ^13^C NMR (100 MHz, DMSO-*d*_6_) *δ* 179.09, 168.23, 155.75, 152.65 (d, *J* = 40.1 Hz), 152.36, 134.97, 134.03, 133.75, 131.23, 130.38, 129.47, 129.39, 128.84, 127.81, 122.03, 121.48, 121.14, 114.68, 108.31, 28.03, 24.22; HRMS (FAB) calc. for C_21_H_13_ClF_3_N_5_O_2_S [M + H]^+^ 492.0509, found: 492.0497.
*4-((3-(2-(4-(3-Chlorophenyl)-2-(trifluoromethyl)thiazol-5-yl)ethyl)-1,2,4-oxadiazol-5-yl)methyl)-1,3-dihydro-2H-benzo[d]imidazol-2-one* (**64**). Compound **64** was synthesized from **30** and **41** according to general procedure G. 69% yield, 96.24% purity, pale yellow solid; ^1^H NMR (400 MHz, DMSO-*d*_6_) *δ* 10.80 (s, 1H), 10.67 (s, 1H), 7.59–7.60 (m, 1H), 7.45–7.53 (m, 3H), 6.81–6.87 (m, 2H), 6.73 (dd, *J* = 6.6, 2.1 Hz, 1H), 4.29 (s, 2H), 3.39 (t, *J* = 7.3 Hz, 2H), 3.08 (t, *J* = 7.3 Hz, 2H); ^13^C NMR (100 MHz, DMSO-*d*_6_) *δ* 178.43, 169.13, 155.77, 151.54, 151.35 (d, *J* = 39.1 Hz), 139.46, 135.51, 133.94, 131.16, 130.38, 129.31, 129.20, 128.86, 127.84, 121.92, 121.13, 120.23 (d, *J* = 269.2 Hz), 114.87, 108.23, 27.82, 27.33, 24.28; HRMS (FAB) calc. for C_22_H_15_ClF_3_N_5_O_2_S [M + H]^+^ 506.0665, found: 506.0667.
*4-((3-((2-(tert-Butyl)-4-(3-chlorophenyl)thiazol-5-yl)methyl)-1,2,4-oxadiazol-5-yl)methyl)-1,3-dihydro-2H-benzo[d]imidazol-2-one* (**65**). Compound **65** was synthesized from **27** and **41** according to general procedure G. 56% yield, 99.36% purity, pale yellow solid; ^1^H NMR (400 MHz, DMSO-*d*_6_) *δ* 10.79 (s, 1H), 10.67 (s, 1H), 7.65–7.66 (m, 1H), 7.58 (td, *J* = 4.2, 2.3 Hz, 1H), 7.40–7.46 (m, 2H), 6.78–6.88 (m, 3H), 4.34 (s, 2H), 4.32 (s, 2H), 1.34 (s, 9H); ^13^C NMR (100 MHz, DMSO-*d*_6_) *δ* 179.05, 178.84, 168.94, 155.76, 149.81, 136.74, 133.78, 130.92, 130.38, 129.41, 128.67, 128.40, 127.54, 127.10, 122.01, 121.13, 114.77, 108.29, 37.90, 30.97, 28.04, 24.17; HRMS (FAB) calc. for C_24_H_22_ClN_5_O_2_S [M + H]^+^ 480.1261, found: 480.1257.
*4-((3-((3-(tert-Butyl)-1-(3-chlorophenyl)-1H-pyrazol-5-yl)methyl)-1,2,4-oxadiazol-5-yl)methyl)-1,3-dihydro-2H-benzo[d]imidazol-2-one* (**66**). Compound **66** was synthesized from **34** and **41** according to general procedure G. 68% yield, 99.57% purity, pale yellow solid; ^1^H NMR (400 MHz, DMSO-*d*_6_) *δ* 10.78 (s, 1H), 10.66 (s, 1H), 7.55–7.56 (m, 1H), 7.38–7.46 (m, 3H), 6.81–6.87 (m, 2H), 6.72 (dd, *J* = 7.1, 1.6 Hz, 1H), 6.18 (s, 1H), 4.30 (s, 2H), 4.19 (s, 2H), 1.21 (s, 9H); ^13^C NMR (100 MHz, DMSO-*d*_6_) *δ* 178.58, 167.86, 162.19, 155.76, 141.07, 137.98, 133.89, 131.22, 130.37, 129.34, 127.87, 124.87, 123.62, 121.90, 121.12, 114.87, 108.23, 105.47, 32.35, 30.75, 27.90, 23.81; HRMS (FAB) calc. for C_24_H_23_ClN_6_O_2_ [M + H]^+^ 463.1649, found: 463.1641.
*7-Methyl-1-((3-((4′-(trifluoromethyl)-[1,1′-biphenyl]-3-yl)methyl)-1,2,4-oxadiazol-5-yl)methyl)-1,7-dihydro-6H-purin-6-one* (**67**). Compound **67** was synthesized from N’-hydroxy-2-(4’-(trifluoromethyl)-[1,1’-biphenyl]-3-yl)acetimidamide and **45** according to general procedure G. 55% yield, 99.91% purity, white solid; ^1^H NMR (400 MHz, DMSO-*d*_6_) *δ* 8.40 (s, 1H), 8.18 (s, 1H), 7.76–7.81 (m, 4H), 7.57–7.61 (m, 2H), 7.42 (t, *J* = 7.8 Hz, 1H), 7.31 (d, *J* = 7.8 Hz, 1H), 5.50 (s, 2H), 4.14 (s, 2H), 3.87 (s, 3H); ^13^C NMR (100 MHz, DMSO-*d*_6_) *δ* 175.97, 169.87, 157.06, 154.05, 147.85, 145.83, 144.40, 139.37, 136.98, 129.91, 129.57, 128.45 (d, *J* = 31.5 Hz), 128.24, 128.01, 126.35, 126.31, 126.27, 126.24, 115.12, 42.08, 33.92, 31.60; HRMS (FAB) calc. for C_23_H_17_F_3_N_6_O_2_ [M + H]^+^ 467.1443, found: 467.1437.
*7-Methyl-1-((3-((4’-(trifluoromethyl)-[1,1’-biphenyl]-4-yl)methyl)-1,2,4-oxadiazol-5-yl)methyl)-1,7-dihydro-6H-purin-6-one* (**68**). Compound **68** was synthesized from N’-hydroxy-2-(4’-(trifluoromethyl)-[1,1’-biphenyl]-4-yl)acetimidamide and **45** according to general procedure G. 49% yield, 99.53% purity, white solid; ^1^H NMR (400 MHz, DMSO-*d*_6_) *δ* 8.40 (s, 1H), 8.19 (s, 1H), 7.84 (d, *J* = 8.2 Hz, 2H), 7.76 (d, *J* = 8.2 Hz, 2H), 7.65 (d, *J* = 8.2 Hz, 2H), 7.37 (d, *J* = 8.2 Hz, 2H), 5.50 (s, 2H), 4.11 (s, 2H), 3.90 (s, 3H); ^13^C NMR (100 MHz, DMSO-*d*_6_) *δ* 175.95, 169.87, 157.06, 154.05, 147.83, 145.85, 144.29, 137.82, 136.39, 130.25, 128.32 (d, *J* = 31.5 Hz), 127.93, 127.79, 126.31, 126.27, 126.25, 115.13, 42.09, 33.96, 31.34; HRMS (FAB) calc. for C_23_H_17_F_3_N_6_O_2_ [M + H]^+^ 467.1443, found: 467.1444.
*7-Methyl-1-((3-(2-(4-methylpiperidin-1-yl)-6-(trifluoromethyl)pyridin-3-yl)-1,2,4-oxadiazol-5-yl)methyl)-1,7-dihydro-6H-purin-6-one* (**69**). Compound **69** was synthesized from **10** and **45** according to general procedure G. 59% yield, 98.20% purity, pale yellow solid; ^1^H NMR (400 MHz, DMSO-*d*_6_) *δ* 8.43 (d, *J* = 7.6 Hz, 1H), 8.39 (s, 1H), 8.20 (s, 1H), 7.46 (d, *J* = 7.9 Hz, 1H), 5.51 (s, 2H), 3.89 (s, 3H), 3.59 (d, *J* = 13.3 Hz, 2H), 2.75 (t, *J* = 11.8 Hz, 2H), 1.52 (d, *J* = 11.7 Hz, 2H), 1.43–1.48 (m, 1H), 1.05–1.14 (m, 2H), 0.85 (d, *J* = 5.9 Hz, 3H); ^13^C NMR (100 MHz, DMSO-*d*_6_) *δ* 175.82, 169.68, 159.10, 157.06, 154.05, 147.79, 146.65, 143.65, 145.80, 129.41, 121.94 (d, *J* = 271.2 Hz), 115.12, 114.59, 50.04, 41.95, 34.00, 33.93, 30.63, 22.25; HRMS (FAB) calc. for C_21_H_21_F_3_N_8_O_2_ [M + H]^+^ 475.1818, found: 475.1809.
*7-Methyl-1-((3-((2-(4-methylpiperidin-1-yl)-6-(trifluoromethyl)pyridin-3-yl)methyl)-1,2,4-oxadiazol-5-yl)methyl)-1,7-dihydro-6H-purin-6-one* (**70**). Compound **70** was synthesized from **13** and **45** according to general procedure G. 61% yield, 99.53% purity, light brown solid; ^1^H NMR (400 MHz, DMSO-*d*_6_) *δ* 8.38 (s, 1H), 8.19 (s, 1H), 7.68 (d, *J* = 7.8 Hz, 1H), 7.38 (d, *J* = 7.3 Hz, 1H), 5.50 (s, 2H), 4.13 (s, 2H), 3.90 (s, 3H), 3.28–3.30 (m, 2H), 2.66 (t, *J* = 12.3 Hz, 2H), 1.56 (d, *J* = 12.8 Hz, 2H), 1.39–1.47 (m, 1H), 1.08–1.19 (m, 2H), 0.85 (d, *J* = 6.4 Hz, 3H); ^13^C NMR (100 MHz, DMSO-*d*_6_) *δ* 175.92, 169.15, 162.33, 157.03, 154.00, 147.77, 145.81, 143.93, 141.17, 127.74, 122.03 (d, *J* = 265.4 Hz), 115.11, 114.70, 50.59, 41.94, 34.16, 33.93, 30.60, 27.92, 22.27; HRMS (FAB) calc. for C_22_H_23_F_3_N_8_O_2_ [M + H]^+^ 489.1974, found: 489.1985.
*7-Methyl-1-((3-(2-(2-(4-methylpiperidin-1-yl)-6-(trifluoromethyl)pyridin-3-yl)ethyl)-1,2,4-oxadiazol-5-yl)methyl)-1,7-dihydro-6H-purin-6-one* (**71**). Compound **71** was synthesized from **16** and **45** according to general procedure G. 60% yield, 99.69% purity, white solid; ^1^H NMR (400 MHz, DMSO-*d*_6_) *δ* 8.40 (s, 1H), 8.20 (s, 1H), 7.79 (d, *J* = 7.3 Hz, 1H), 7.32 (d, *J* = 7.3 Hz, 1H), 5.50 (s, 2H), 3.91 (s, 3H), 3.26–3.30 (m, 2H), 3.04–3.08 (m, 2H), 2.95–2.99 (m, 2H), 2.66 (td, *J* = 12.1, 1.7 Hz, 2H), 1.59 (d, *J* = 12.3 Hz, 2H), 1.40–1.49 (m, 1H), 1.10–1.19 (m, 2H), 0.86 (d, *J* = 6.9 Hz, 3H); ^13^C NMR (100 MHz, DMSO-*d*_6_) *δ* 175.73, 170.22, 162.38, 157.08, 154.02, 147.80, 145.79, 142.95 (d, *J* = 33.4 Hz), 139.73, 131.85, 122.19 (d, *J* = 271.2 Hz), 115.12, 114.66, 50.77, 41.96, 34.37, 33.93, 30.71, 27.97, 24.96, 22.28; HRMS (FAB) calc. for C_23_H_25_F_3_N_8_O_2_ [M + H]^+^ 503.2131, found: 503.2123.
*1-((3-(4-(3-Chlorophenyl)-2-(trifluoromethyl)thiazol-5-yl)-1,2,4-oxadiazol-5-yl)methyl)-7-methyl-1,7-dihydro-6H-purin-6-one* (**72**). Compound **72** was synthesized from **21** and **45** according to general procedure G. 76% yield, 99.89% purity, white solid; ^1^H NMR (400 MHz, DMSO-*d*_6_) *δ* 8.38 (s, 1H), 8.21 (s, 1H), 7.74 (t, *J* = 1.8 Hz, 1H), 7.60 (dt, *J* = 7.8, 1.3 Hz, 1H), 7.45–7.48 (m, 1H), 7.36 (t, *J* = 8.0 Hz, 1H), 5.60 (s, 2H), 3.90 (s, 3H); ^13^C NMR (100 MHz, DMSO-*d*_6_) *δ* 176.91, 161.91, 157.08, 155.58 (d, *J* = 40.1 Hz), 154.99, 154.01, 147.70, 145.85, 134.41, 133.32, 130.51, 130.14, 129.69, 128.76, 123.17, 119.68 (d, *J* = 264.4 Hz), 115.09, 41.95, 33.95; HRMS (FAB) calc. for C_19_H_11_ClF_3_N_7_O_2_S [M + H]^+^ 494.0414, found: 494.0426.
*1-((3-((4-(3-Chlorophenyl)-2-(trifluoromethyl)thiazol-5-yl)methyl)-1,2,4-oxadiazol-5-yl)methyl)-7-methyl-1,7-dihydro-6H-purin-6-one* (**73**). Compound **73** was synthesized from **26** and **45** according to general procedure G. 48% yield, 99.23% purity, pale yellow solid; ^1^H NMR (400 MHz, DMSO-*d*_6_) *δ* 8.38 (s, 1H), 8.20 (s, 1H), 7.67 (t, *J* = 1.6 Hz, 1H), 7.56 (dt, *J* = 7.0, 1.7 Hz, 1H), 7.44–7.50 (m, 2H), 5.52 (s, 2H), 4.59 (s, 2H), 3.89 (s, 3H); ^13^C NMR (100 MHz, DMSO-*d*_6_) *δ* 177.59, 173.55, 165.98, 159.79, 157.09, 154.00, 152.46, 147.73, 145.94, 135.37, 134.01, 130.72, 129.67, 128.77, 127.80, 121.47 (d, *J* = 63.0 Hz), 115.11, 41.95, 33.96, 24.12; HRMS (FAB) calc. for C_20_H_13_ClF_3_N_7_O_2_S [M + H]^+^ 508.0570, found: 508.0564.
*1-((3-(2-(4-(3-Chlorophenyl)-2-(trifluoromethyl)thiazol-5-yl)ethyl)-1,2,4-oxadiazol-5-yl)methyl)-7-methyl-1,7-dihydro-6H-purin-6-one* (**74**). Compound **74** was synthesized from **30** and **45** according to general procedure G. 83% yield, 99.39% purity, white solid; ^1^H NMR (400 MHz, DMSO-*d*_6_) *δ* 8.38 (s, 1H), 8.19 (s, 1H), 7.60–7.61 (m, 1H), 7.42–7.53 (m, 3H), 5.48 (s, 2H), 3.88 (s, 3H), 3.38 (t, *J* = 7.3 Hz, 2H), 3.11 (t, *J* = 7.3 Hz, 2H); ^13^C NMR (100 MHz, DMSO-*d*_6_) *δ* 176.00, 169.29, 157.06, 153.98, 151.54, 151.18, 147.74, 145.79, 139.31, 135.49, 133.93, 131.10, 129.19, 128.86, 127.81, 120.23 (d, *J* = 269.2 Hz), 115.11, 42.01, 33.87, 27.13, 24.16; HRMS (FAB) calc. for C_21_H_15_ClF_3_N_7_O_2_S [M + H]^+^ 522.0727, found: 522.0730.
*1-((3-((2-(tert-Butyl)-4-(3-chlorophenyl)thiazol-5-yl)methyl)-1,2,4-oxadiazol-5-yl)methyl)-7-methyl-1,7-dihydro-6H-purin-6-one* (**75**). Compound **75** was synthesized from **27** and **45** according to general procedure G. 72% yield, 99.75% purity, pale yellow solid; ^1^H NMR (400 MHz, DMSO-*d*_6_) *δ* 8.40 (s, 1H), 8.20 (s, 1H), 7.65–7.66 (m, 1H), 7.54–7.57 (m, 1H), 7.40–7.41 (m, 2H), 5.53 (s, 2H), 4.35 (s, 2H), 3.90 (s, 3H), 1.34 (s, 9H); ^13^C NMR (100 MHz, DMSO-*d*_6_) *δ* 179.12, 176.40, 169.11, 157.09, 154.05, 149.94, 147.79, 145.82, 136.67, 133.75, 130.90, 128.64, 128.42, 127.53, 126.76, 115.13, 42.13, 37.90, 33.95, 30.95, 24.05; HRMS (FAB) calc. for C_23_H_22_ClN_7_O_2_S [M + H]^+^ 496.1322, found: 496.1307.
*1-((3-((3-(tert-Butyl)-1-(3-chlorophenyl)-1H-pyrazol-5-yl)methyl)-1,2,4-oxadiazol-5-yl)methyl)-7-methyl-1,7-dihydro-6H-purin-6-one* (**76**). Compound **76** was synthesized from **34** and **45** according to general procedure G. 76% yield, 99.29% purity, white solid; ^1^H NMR (400 MHz, DMSO-*d*_6_) *δ* 8.38 (s, 1H), 8.20 (s, 1H), 7.55–7.56 (m, 1H), 7.36–7.43 (m, 3H), 6.15 (s, 1H), 5.49 (s, 2H), 4.21 (s, 2H), 3.90 (s, 3H), 1.19 (s, 9H); ^13^C NMR (100 MHz, DMSO-*d*_6_) *δ* 176.17, 168.00, 162.16, 157.06, 154.02, 147.77, 145.81, 141.04, 137.79, 133.88, 131.23, 127.90, 124.87, 123.67, 115.12, 105.41, 42.01, 33.93, 32.33, 30.70, 23.70; HRMS (FAB) calc. for C_23_H_23_ClN_8_O_2_ [M + H]^+^ 479.1711, found: 479.1702.

### 3.2. In Vitro Assay

#### Fluorescence Imaging Plate Reader Assay

Chinese hamster ovary (CHO) cells stably expressing human TRPV1 (hTRPV1) (AddexBio, San Diego, CA, USA), human embryonic kidney (HEK293) cells stably expressing human TRPA1 (hTRPA1) (Eurofins, Luxembourg), and HEK293 cells (ATCC, CRL-1573) transiently transfected with pcDNA 3.1 plasmids of mouse TRPA1 (mTRPA1)/rat TRPV1 (rTRPV1) using Neon Nucleofector (Thermo Fisher Scientific, Waltham, MA, USA) were seeded in clear-bottom black 96-well plates (Corning, NY, USA) and grown to confluency. On the assay day, cells were loaded with 2 μM of the membrane-permeant Ca^2+^ indicator Fluo-4 AM (Thermo Fisher Scientific, Waltham, MA, USA), 0.02% pluronic F-127 (Thermo Fisher Scientific, Waltham, MA, USA), and 1 mM probenecid (Thermo Fisher Scientific, Waltham, MA, USA) in Opti-MEM serum-free media (Thermo Fisher Scientific, Waltham, MA, USA) for 1 h in the dark. The media was then replaced with an assay buffer containing test compounds, which included (in mM) 140 NaCl, 5 KCl, 2 CaCl_2_, 2 MgCl_2_, and 10 HEPES, adjusted to pH 7.4 with NaOH (all chemicals from Sigma-Aldrich, St. Louis, MO, USA), and incubated for another hour in the dark. Solutions containing test compounds and standard agonists were added to each well, and the plate was placed in the EnSpire Multimode Plate Reader (PerkinElmer, Waltham, MA, USA). Fluorescence signals were recorded at a rate of 1 plate per minute with a 100 ms exposure time. Data were normalized to the standard agonist signal obtained when treated with buffer without test compounds. To verify the appropriate transfection of each TRP channel, calcium signals triggered by the application of standard agonists were measured in the same batches (capsaicin for rTRPV1 and hTRPV1; cinnamaldehyde for mTRPA1 and hTRPA1). All experiments were performed in triplicate, and data points represent means ± S.E.M. IC_50_ measurements and curve fitting were conducted using GraphPad Prism 10.2.3 software.

### 3.3. In Vivo Assay

#### 3.3.1. Animals

ICR male mice weighing 23–25 g were purchased from Samtako Korea (Osan, Korea). ICR mice were housed four per cage in a room with 12 h light–dark cycles. The temperature and relative humidity of the room were maintained at 22 ± 2 °C and 50 ± 5%, respectively. Food and water were available ad libitum. The procedures for animal testing were approved by Medifron Animal Care and Use Committees (Approval number; Medifron 2019-9). Efforts were made to minimize animal suffering and to reduce the number of animals used.

#### 3.3.2. Formalin Test

Mice were randomly assigned to five groups (four per group) and single-dose drugs were administered by intraperitoneal injection. The formalin-induced licking paw test was modified from the method described by Dubuisson and Dennis [44,45]. Each mouse was acclimated to an acrylic observation chamber for at least 30 min before the injection of formalin. Twenty microliters of 2% formalin was injected subcutaneously into the right side of the hind paw. Each mouse was then placed in an individual clear plastic observational chamber (15 × 15 × 15), and the pain response was recorded for a period of 30 min. The summation of time (in seconds) spent in licking and biting responses of the injected paw during each 5 min block was measured as an indicator of the pain response. The first period (early phase) was recorded 0–5 min after the injection of formalin, and the second period (late phase) was recorded 20–30 min after the injection. The test compound was administered intraperitoneally 30 min before the formalin injection at three different doses: 10, 20, 50, and 100 mg/Kg. The vehicle was DMSO/Cremophor EL/D.W. (10/10/80). The data were expressed as the mean ± standard error of the mean (SEM). Statistical analysis was assessed by one-way analysis of variance (ANOVA) with Bonferroni’s post-hoc test. A *p*-value < 0.05 was considered statistically significant.

## 4. Conclusions

Effectively managing pain continues to be a significant challenge in medicine, emphasizing the necessity for developing new therapeutic agents. Ion channels such as transient receptor potential ankyrin 1 (TRPA1) and vanilloid 1 (TRPV1) are pivotal in pain perception. Simultaneously targeting both TRPA1 and TRPV1 with dual antagonists presents a promising method for achieving pain relief.

To discover dual antagonists targeting both TRPA1 and TRPV1 simultaneously as new therapeutic agents, a series of hybrid analogs of TRPA1 and TRPV1 antagonists were designed and synthesized to discover novel therapeutic agents for pain. Among them, compound **50** exhibited dual-acting antagonism to TRPA1 and TRPV1 with IC_50_ values of 1.42, 2.84, 2.13 and 5.02 μM for hTRPA1, mTRPA1, hTRPV1, and rTRPV1, respectively. In the formalin test, compound **50** demonstrated dose-dependent analgesic activity with an ED_50_ of 85.9 mg/kg in the phase 1 response and 21.6 mg/kg in the phase 2 response, respectively, and was able to inhibit pain behavior completely at a dose of 100 mg/kg. In this study, we present the discovery and characterization of a novel dual TRPA1/TRPV1 antagonist, rationally designed using a hybrid approach distinct from two previously reported dual antagonists, demonstrating potential as a therapeutic agent for pain management.

## Figures and Tables

**Table 1:** In vitro activities of 4-(1,2,4-oxadiazol-5-yl) benzimidazolone analogs.

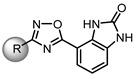					
		**% Inhibition ^1^**			
**Compound**	**R**	**hTRPA1**	**mTRPA1**	**hTRPV1**	**rTRPV1**
**AM-0902**		100%	125%		
**BCTC**				100%	100%
**46**	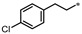	33%	27%	7%	10%
**47**	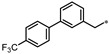	38%	26%	15%	17%
**48**	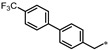	NE	21%	45%	33%
**49**	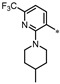	40%	14%	12%	15%
**50**	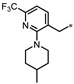	60%	44%	52%	48%
**51**	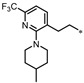	NE	21%	20%	11%
**52**	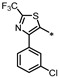	NE	40%	8%	NE
**53**	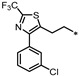	NE	48%	13%	NE
**54**	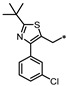	NE	4%	43%	25%
**55**	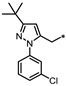	29%	60%	28%	NE

^1^ Percent inhibition toward the activation of an agonist at 10 μM compound. NE—no effect.

**Table 2:** In vitro activities of 4-((1,2,4-oxadiazol-5-yl)methyl) benzimidazolone analogs.

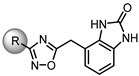					
		**% Inhibition ^1^**			
**Compound**	**R**	**hTRPA1**	**mTRPA1**	**hTRPV1**	**rTRPV1**
**56**	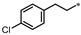	32%	29%	34%	NE
**57**	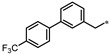	23%	24%	45%	29%
**58**	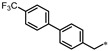	33%	15%	31%	20%
**59**	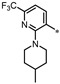	40%	24%	13%	1%
**60**	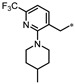	22%	32%	24%	29%
**61**	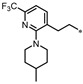	29%	25%	64%	62%
**62**	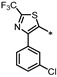	NE	16%	23%	28%
**63**	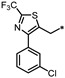	17%	17%	51%	30%
**64**	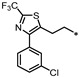	NE	20%	21%	24%
**65**	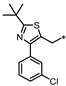	24%	23%	69%	40%
**66**	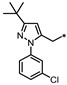	34%	55%	77%	46%

^1^ percent inhibition toward the activation of agonist at 10 μM compound. NE—no effect.

**Table 3:** In vitro activities of 4-((1,2,4-oxadiazol-5-yl)methyl) 7-methyl purinone analogs.

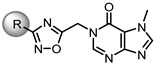					
		**% Inhibition ^1^**			
**Compound**	**R**	**hTRPA1**	**mTRPA1**	**hTRPV1**	**rTRPV1**
**67**	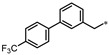	NE	16%	NE	NE
**68**	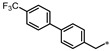	35%	35%	7%	18%
**69**	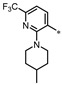	NE	16%	16%	NE
**70**	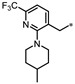	9%	34%	22%	NE
**71**	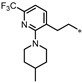	11%	28%	NE	NE
**72**	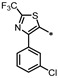	35%	46%	31%	4%
**73**	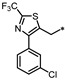	NE	22%	10%	NE
**74**	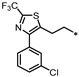	5%	14%	3%	NE
**75**	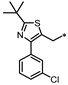	11%	43%	14%	NE
**76**	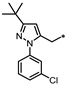	7%	50%	7%	NE

^1^ Percent inhibition toward the activation of agonist at 10 μM compound. NE—no effect.

## Data Availability

Data is contained within the article.
